# Combined venetoclax and alvocidib in acute myeloid leukemia

**DOI:** 10.18632/oncotarget.22284

**Published:** 2017-11-03

**Authors:** James Bogenberger, Clifford Whatcott, Nanna Hansen, Devora Delman, Chang-Xin Shi, Wontak Kim, Hillary Haws, Katherine Soh, Ye Sol Lee, Peter Peterson, Adam Siddiqui-Jain, Steven Weitman, Keith Stewart, David Bearss, Ruben Mesa, Steven Warner, Raoul Tibes

**Affiliations:** ^1^ Mayo Clinic, Scottsdale, AZ, USA; ^2^ Tolero Pharmaceuticals, Lehi, UT, USA; ^3^ NYU School of Medicine, New York, NY, USA

**Keywords:** acute myeloid leukemia (AML), alvocidib/flavopiridol, venetoclax/ABT-199, BCL-2

## Abstract

More effective treatment options for elderly acute myeloid leukemia (AML) patients are needed as only 25–50% of patients respond to standard-of-care therapies, response duration is typically short, and disease progression is inevitable even with some novel therapies and ongoing clinical trials. Anti-apoptotic BCL-2 family inhibitors, such as venetoclax, are promising therapies for AML. Nonetheless, resistance is emerging. We demonstrate that venetoclax combined with cyclin-dependent kinase (CDK) inhibitor alvocidib is potently synergistic in venetoclax-sensitive and -resistant AML models *in vitro*, *ex vivo* and *in vivo*. Alvocidib decreased MCL-1, and/or increased pro-apoptotic proteins such as BIM or NOXA, often synergistically with venetoclax. Over-expression of BCL-XL diminished synergy, while knock-down of BIM almost entirely abrogated synergy, demonstrating that the synergistic interaction between alvocidib and venetoclax is primarily dependent on intrinsic apoptosis. CDK9 inhibition predominantly mediated venetoclax sensitization, while CDK4/6 inhibition with palbociclib did not potentiate venetoclax activity. Combined, venetoclax and alvocidib modulate the balance of BCL-2 family proteins through complementary, yet variable mechanisms favoring apoptosis, highlighting this combination as a promising therapy for AML or high-risk MDS with the capacity to overcome intrinsic apoptosis mechanisms of resistance. These results support clinical testing of combined venetoclax and alvocidib for the treatment of AML and advanced MDS.

## INTRODUCTION

Targeting of intrinsic apoptosis via inhibition of B-Cell Lymphoma 2 (BCL-2) family proteins is a promising therapeutic strategy. BCL-2-selective inhibitor venetoclax (ABT-199) has demonstrated clinical activity in chronic lymphocytic leukemia (CLL) and has recently received FDA-approval for some relapsed CLL patients [1]. Importantly, BCL-2 family proteins are also therapeutic targets in acute myeloid leukemia (AML) [2–7] and high-risk myelodysplastic syndrome (MDS) where increases in anti-apoptotic BCL-2 family proteins are associated with disease progression and apoptotic resistance [8–12]. Clinical activity of venetoclax monotherapy is modest in AML [13], which may partially be explained by the genetic heterogeneity of AML, and more importantly by the heterogeneous and concurrent expression of multiple anti-apoptotic BCL-2 family proteins in AML, and the selectivity of venetoclax for BCL-2 [14]. Consequently, therapeutics targeting complementary BCL-2 family proteins may increase the efficacy of venetoclax. Preclinical data demonstrate that BCL-2 family inhibition synergizes with hypomethylating agents (HMAs) [14–16] and, although the precise mechanism of synergy remains incompletely characterized, HMAs may down-regulate MCL-1 [16]. Initial clinical trials examining venetoclax with HMAs in previously untreated elderly AML patients have reported impressive overall response rates of > 75% [17], although clinical resistance is emerging. Putative mechanisms of venetoclax resistance include compensatory up-regulation, increased stability, or altered function of MCL-1 [18–20]. MCL-1 plays a role in intrinsic resistance to BCL-2 inhibitors by alternatively sequestering BIM dissociated from BCL-2 by BCL-2 inhibitors [21–23]. MCL-1 inhibition or genetic knock-down is known to potentiate BCL-2 inhibitors [7, 14, 24]. The cyclin-dependent kinase (CDK) inhibitor alvocidib (flavopiridol) reduces MCL-1 in hematologic malignancies [25–27]. Alvocidib is a potent inhibitor of CDK9, the core catalytic component of the P-TEFb complex [28–30]. Transcriptional repression of short half-life oncoproteins, such as MCL-1, can potently induce apoptosis; however, additional anti-apoptotic proteins, such as BCL-2, can functionally counter this repression by sequestering pro-apoptotic BH3-only proteins or blocking BAX/BAK dimerization. Increased pro-apoptotic BH3-only proteins such as BIM have also been observed in response to alvocidib treatment [31]. We hypothesized that alvocidib would synergize with BCL-2 inhibitor venetoclax in AML through complementary mechanisms, namely decreased MCL-1 and increased BIM, to modulate the overall balance of anti- and pro-apoptotic BCL-2 family proteins in favor of apoptosis induction.

## RESULTS

### Alvocidib potentiates venetoclax anti-leukemic activity in both venetoclax-sensitive and –resistant AML cells

**Figure 1:**
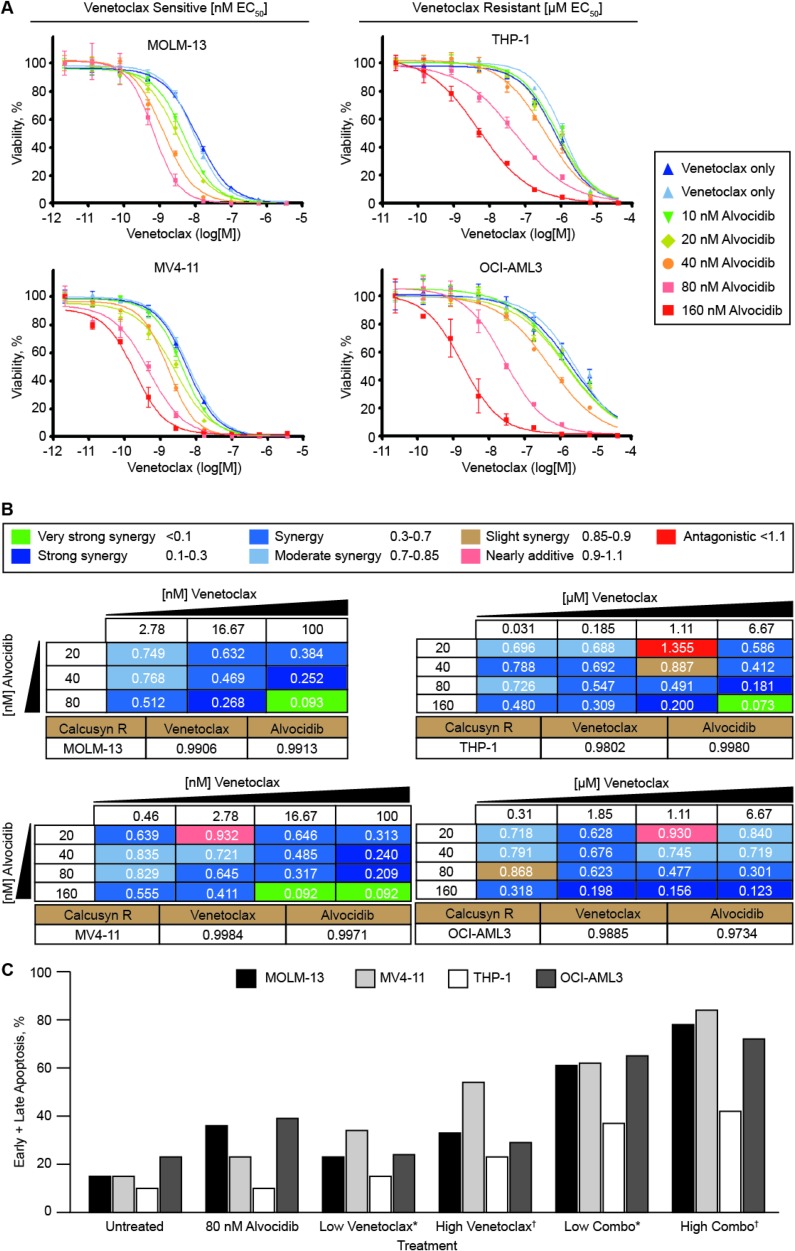
Alvocidib potentiates venetoclax anti-leukemic activity in both venetoclax -sensitive and –resistant AML cells (**A–B**) combination drug dose response assays with venetoclax and alvocidib *in vitro* were assessed in duplicate biological experiments, each containing four technical replicate data points for every dose/dose combination analyzed. Data represent mean ± SEM. The indicated AML cell lines were dosed with venetoclax or alvocidib as single-agent, and in combination, and incubated for 96 hours before determining relative cell number with ATP-based reagent CellTiter Glo. (A) leftward shifts toward lower doses of venetoclax demonstrate dose-dependent venetoclax fold-sensitization by alvocidib. (B) Combination Index (CI) values were calculated with CalcuSyn Software, and are shown for distinct dose combinations of venetoclax and alvocidib. CalcuSyn *R* values corresponding to single-agent dose curves are shown for each cell line below CI value tables. (**C**) AML cell lines were treated for 24 hours with 80 nM alvocidib, and a low dose or high dose of venetoclax, each alone and in combination, prior to harvesting for flow cytometry quantification of annexin V and propidium iodide permeability as a measurement of apoptosis. For venetoclax -sensitive cells MOLM-13 and MV4-11, ^*^2.5 and ^†^10 nM were used, while for venetoclax -resistant cell lines THP-1 and OCI-AML3, ^*^0.25 and ^†^1 µM venetoclax were used. Quantification from a representative experiment is shown graphically, and apoptosis results were confirmed in biological replicate experiments using one venetoclax -sensitive cell line (MOLM-13) and one venetoclax -resistant cell line (THP-1).

To assess the potential for synergy between venetoclax and alvocidib in venetoclax -sensitive AML cells *in vitro*, we selected MOLM-13 and MV4-11 cell lines, which have low single-agent venetoclax half-maximal effective concentration (EC_50_) values (in assays assessing relative cell number) of 9.0 ± 1.6 and 7.8 ± 2.1 nM, respectively. These low nM venetoclax EC_50_ values are consistent with values for primary AML samples sensitive to venetoclax [7]. THP-1 and OCI-AML3 were selected to model venetoclax -resistant AML cells, exhibiting higher single-agent venetoclax EC_50_ values of 0.9 ± 0.2 and 2.3 ± 0.4 µM, respectively, 100- to 295-fold greater than venetoclax -sensitive cells. Addition of alvocidib resulted in potent dose-dependent reduction of venetoclax EC_50_ values in both venetoclax -sensitive and –resistant cells (Figure 1A). venetoclax EC_50_ fold-sensitization by alvocidib was greatest in venetoclax -resistant cells, with 80 nM alvocidib resulting in an 18.3 ± 0.1 (*p* = 0.0039) and 77.7 ± 1.2 (*p* = 0.0069) venetoclax EC_50_ fold-shift for THP-1 and OCI-AML3, respectively. Importantly, alvocidib shifted the absolute EC_50_ values of venetoclax -resistant cells from µM to low nM doses (30 to 50 nM) similar to that of sensitive cells/primary samples. Fold-sensitization at 80 nM alvocidib was 14.5 ± 0.8 (*p* = 0.028) and 10.1 ± 3.4 (*p* = 0.17) in MOLM-13 and MV4-11, respectively. At clinically-achievable plasma concentrations of 80 and 160 nM alvocidib, synergy (expressed as Combination Index (CI) values) was observed with all clinically-achievable doses of venetoclax tested, in all cell lines examined (Figure 1B). To confirm that synergistic effects of combined venetoclax and alvocidib culminate in increased apoptosis, as opposed to only cytostatic effects from putatively inhibiting cell cycle CDKs, we analyzed Annexin V levels and propidium iodide permeability by flow cytometry. In all cells examined, we observed an increase in early and late apoptotic cells in response to the combination beyond the additive effects of either single-agent (Figure 1C). In parallel, we assessed cell cycle distributions and found that 80 nM alvocidib resulted only in a moderate proportional increase in G1, with corresponding decreases in S and G2, in three of four cell lines analyzed (median increase 28 ± 5%); however, 80 nM alvocidib did not significantly alter cell cycle distribution in OCI-AML3 (Supplementary Figure 1).

### Correlation of BCL-2 family proteins with alvocidib/venetoclax activity

**Figure 2:**
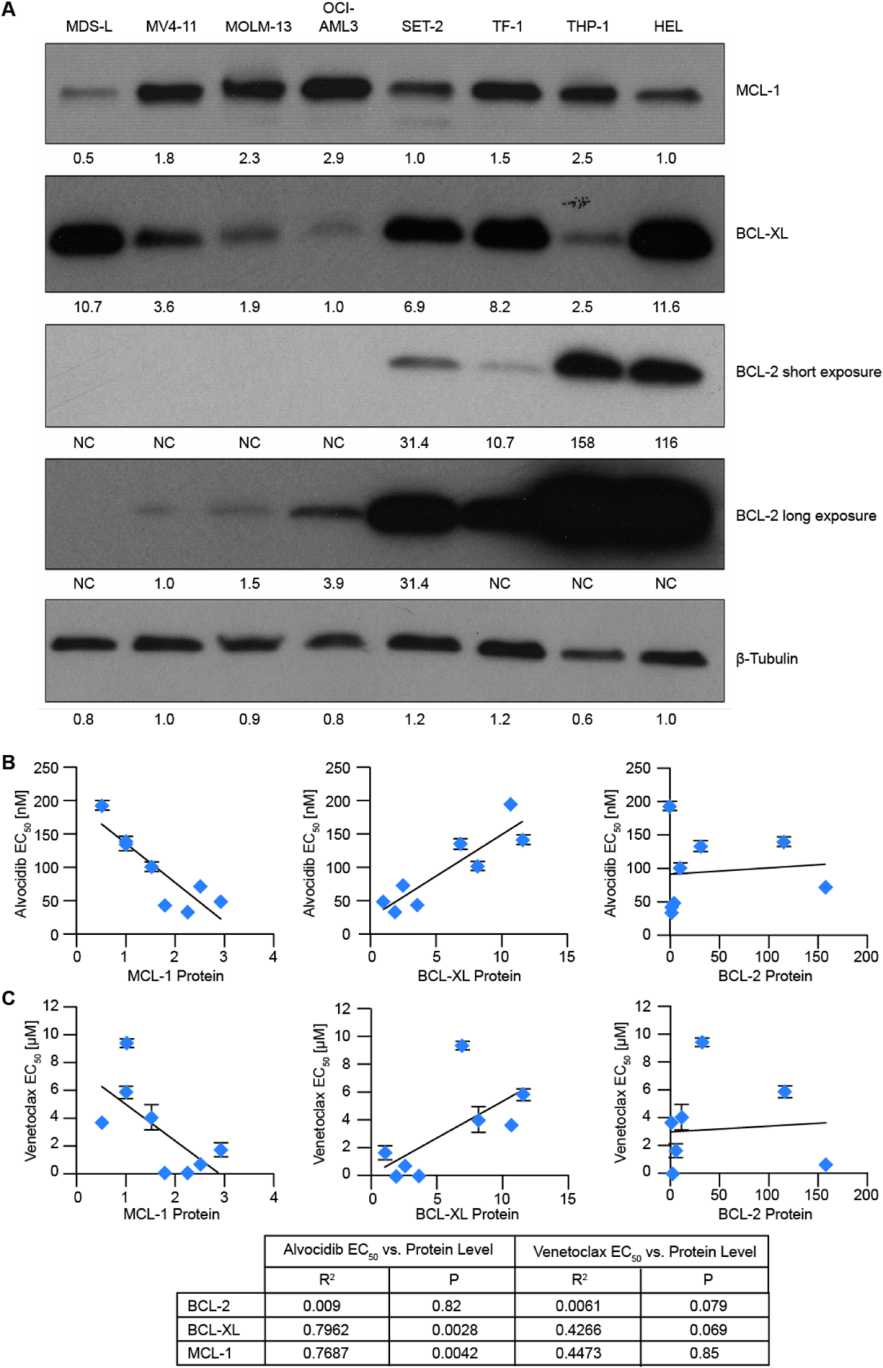
Correlation of BCL-2 family proteins with alvocidib and venetoclax activity (**A**) lysates were prepared from untreated AML cell lines, and levels of the indicated anti-apoptotic BCL-2 family proteins measured by western blot. Image J densitometry software was used to quantify bands and values normalized to β-tubulin. (**B** and **C**), relative protein levels of MCL-1, BCL-XL and BCL-2 were plotted against alvocidib EC_50_ values (B) or venetoclax EC_50_ values (C) determined from duplicate biological experiments. Regression analysis was used to determine R^2^ and *P* values.

To determine whether anti-apoptotic BCL-2 family members correlate with single-agent alvocidib anti-leukemic activity, we initially quantified baseline protein levels of BCL-2, BCL-XL and MCL-1 in untreated cells. MCL-1 protein was relatively homogenous, differing by a median of 1.7 ± 0.8-fold. In contrast, BCL-2 levels were highly variable, differing by 158-fold between the lowest and highest expressing cells. BCL-XL protein expression was also variable spanning an 11.6-fold range (Figure 2A). Relative protein levels were then plotted against single-agent alvocidib EC_50_ values from cell viability assays. BCL-2 levels did not correlate with alvocidib activity, while BCL-XL levels positively correlated, and MCL-1 levels negatively correlated with alvocidib activity (Figure 2B). BCL-2, BCL-XL and MCL-1 protein levels did not significantly correlate with venetoclax single-agent activity in this panel of AML cell lines (Figure 2C).

In contrast to the potent alvocidib sensitization resulting in clinically meaningful 30–50 nM venetoclax EC_50_ values observed in venetoclax-resistant cells with low levels of BCL-XL (BCL-XL^Low^), venetoclax-resistant cells with high levels of BCL-XL (BCL-XL^High^) exhibited EC_50_ values that were not clinically meaningful ranging from 1.7 to 4.7 µM (median 3.4 ± 1.2 µM). ABT-737 (navitoclax), a drug that inhibits BCL-XL and BCL-W in addition to BCL-2, was used to examine the role of these additional BCL-2 family members in alvocidib synergy. Combined with alvocidib, EC_50_ values for ABT-737 were lower than that of venetoclax in BCL-XL^High^ cells (Supplementary Figure 2A, upper panel). EC_50_ fold-sensitization by alvocidib was greater for ABT-737 than venetoclax in BCL-XL^High^ cells (Supplementary Figure 2A, lower panel). In contrast, BCL-XL^Low^ cells (both venetoclax-sensitive and -resistant) were equally, or more, sensitive to single-agent venetoclax than ABT-737 (Supplementary Figure 2B). Knock-down of BCL-XL significantly reduced venetoclax EC_50_ values as compared to non-silencing (NS) siRNA (Supplementary Figure 2C–2D). BCL-XL was over-expressed with a lentiviral construct in both venetoclax-sensitive (MOLM-13) and venetoclax-resistant (THP-1) BCL-XL^Low^ cells, which rendered these cells more resistant to single-agent venetoclax (from nM to µM doses) (Supplementary Figure 2E). Similar to BCL-XL^High^ cells, although sensitization was observed in BCL-XL over-expressing cells, the resultant venetoclax EC_50_ values were no longer clinically meaningful (Supplementary Figure 2F).

### BCL-2 family proteins altered by alvocidib

**Figure 3:**
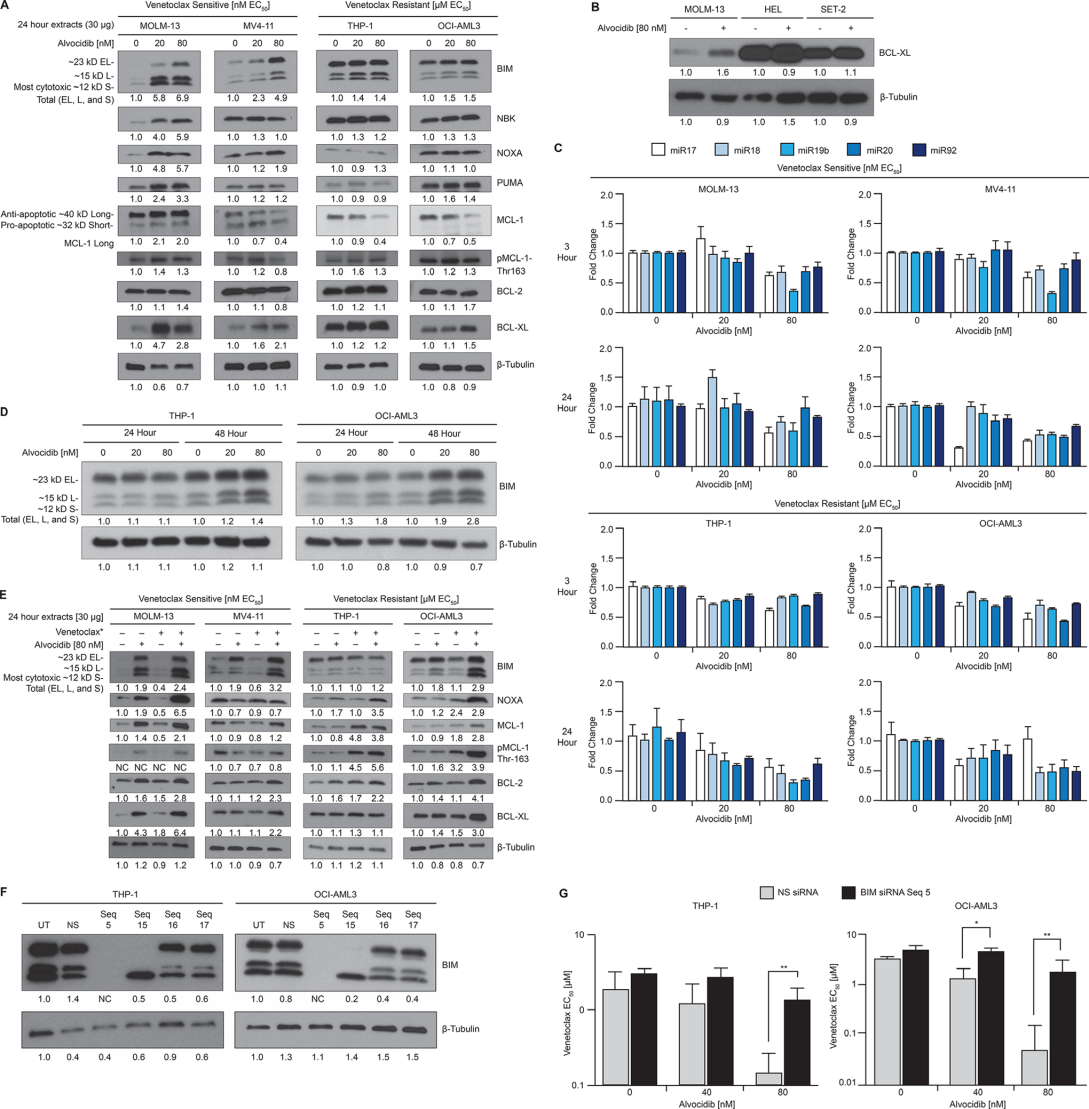
BCL-2 family proteins altered by alvocidib (**A**) AML cell lines were treated with 20 or 80 nM single-agent alvocidib for 24 hours, and BCL-2 family proteins quantified by western blot of duplicate biological extracts. (**B**) AML cell lines with low (MOLM-13) or high (HEL and SET-2) BCL-XL levels were treated with 80 nM alvocidib and resolved on the same gel to facilitate quantitative comparison across these cell lines. (**C**) After treatment with the alvocidib doses indicated on the x-axis, RNA extracts were prepared at 3 hours (upper panel) or 24 hours (lower panel), and the microRNAs indicated were measured by qPCR. Values shown are mean ± SEM of technical replicates and are representative of three biological replicate experiments. (**D**) THP-1 and OCI-AML3 were treated with 20 or 80 nM single-agent alvocidib for 24 and 48 hours, and BIM quantified by western blot. (**E**) AML cell lines were treated with 80 nM alvocidib and ^*^2.5 nM for venetoclax-sensitive lines (MOLM-13 and MV4-11) or ^*^0.25 µM for venetoclax -resistant lines (THP-1 and OCI-AML3), as single-agent and in combination and BCL-2 family proteins quantified by western blot of duplicate biological extracts. (**F**) OCI-AML3 and THP-1 cell lysates prepared after treatment with non-silencing (NS) or BIM siRNA were resolved by gel electrophoresis to quantify the level of specific BIM knock-down. (**G**) BIM siRNA sequence 5 (the only sequence tested that effectively knocked-down all BIM isoforms) was compared against NS siRNA in cells treated with combined venetoclax and alvocidib in duplicate biological experiments. venetoclax EC_50_ values are plotted on a log10 scale at increasing concentrations of alvocidib. Data represent average ± STD. *P* values were calculated using a two-tailed Student’s *T*-test. ^*^P value < 0.05, ^**^P value < 0.01. A–B & D–F, densitometry values shown were calculated using Image J software and normalized to β-Tubulin.

To examine effects of alvocidib on the BCL-2 family protein landscape, we treated venetoclax-sensitive and –resistant AML cells with ascending doses and assessed total protein. Consistent with previous reports in other hematologic malignancies [25–27], we observed a dose-dependent reduction of ∼40 kD anti-apoptotic isoform MCL-1_Long_ in three of four AML cell lines. Increased BIM was observed in all four cell lines in response to alvocidib, although the increase was more pronounced in venetoclax-sensitive cells. MOLM-13 was unique in that we did not observe a reduction in MCL-1_Long_, but instead observed a dose-dependent induction of all BH3-only proteins measured (NBK, NOXA, PUMA and BIM). BCL-2 was not significantly changed by alvocidib treatment. Conversely, BCL-XL was variably induced by alvocidib in all four cell lines (Figure 3A). However, the induced level of BCL-XL in BCL-XL^Low^ MOLM-13 cells was significantly lower than baseline levels in BCL-XL^High^ cells (Figure 3B). MCL-1 protein stability is regulated via complex phosphorylation by multiple kinases including CDK1 and -2 [32, 33]. Signaling via ERK, JNK, GSK3 and CDK2, converge on Thr-163 phosphorylation of MCL-1, which increases MCL-1 stability and thereby plays a role in resistance to BCL-2 inhibitors [34]. To investigate whether alvocidib affects MCL-1 protein stability through putative CDK1/2 inhibition, in addition to MCL-1 transcriptional down-regulation by CDK9 inhibition, we measured MCL-1 Thr-163 phosphorylation levels. Alvocidib did not significantly decrease MCL-1 Thr-163 phosphorylation in two of three cell lines where decreases in total MCL-1 were observed (Figure 3A). Published studies suggest that the polycistronic microRNA (miRNA) miR-17–92 negatively regulates BIM [35, 36], thus we measured five miRNAs derived from the miR-17–92 cluster in response to alvocidib treatment. Relative to control miRNAs, all five miRNAs decreased in a dose- and time-dependent manner (Figure 3C). We were surprised to observe miR-17–92 decreases in THP-1 and OCI-AML3, where potent increases in BIM protein were not observed at 24 hours. Thus, we performed western blots at 48 hours in these two cell lines and observed a more delayed increase of BIM (Figure 3D). Next we examined BCL-2 family proteins after 24 hour combination treatment, as compared to single-agent and untreated controls. Three of four cell lines showed increases in either BIM or NOXA with the combination treatment. Surprisingly, we observed variable increases in MCL-1, BCL-2, and BCL-XL with combination treatment in all cell lines. The observed increase in MCL-1 corresponded to increased Thr-163 phosphorylation, which could primarily be attributed to single-agent venetoclax treatment in venetoclax-resistant cells, but not in venetoclax-sensitive cells (Figure 3E).

The functional importance of reducing/inhibiting MCL-1 for the anti-leukemic activity of BCL-2 inhibitors is well established, including a role in sequestration of BIM liberated by venetoclax [22]. BIM is a critical effector protein of the intrinsic apoptotic pathway with a capacity to act as a dual sensitizer and activator of MOMP. Thus, we sought to evaluate the functional role of BIM, specifically with regard to synergy between alvocidib and venetoclax. Knock-down of BIM prior to alvocidib and venetoclax combination treatment potently abrogated synergy compared to NS siRNA. BIM knock-down abrogated 97% (*p* = 0.0066) and 85% (*p* = 0.0075) of the venetoclax fold-sensitization induced by 80 nM alvocidib in OCI-AML3 and THP-1 cells, respectively. Mean combination venetoclax EC_50_ values were 1.8 and 1.3 µM with BIM siRNA, versus 0.05 and 0.14 µM with NS control siRNA, demonstrating that synergy between venetoclax and alvocidib is almost entirely dependent on the intrinsic apoptotic pathway (Figure 3F–3G).

### Pharmacological dissection of CDK isoforms contributing to venetoclax sensitization

To discern the contributions of different CDK isoforms toward venetoclax sensitization, we examined CDK inhibitors with putatively distinct inhibitory profiles (LDC067, palbociclib, LY2857785, seliciclib, NU6102, and Ro-3306), as well as a BRD4 inhibitor (JQ1) to more specifically assess P-TEFb. We compared single-agent activity of these compounds in a panel of ten AML cell lines with diverse molecular, cytogenetic and lineage backgrounds (MDS-L, HL-60, OCI-AML2, OCI-AML3, MV4-11, MOLM-13, THP-1, TF-1, HEL, and SET-2). Correlation of each compound with alvocidib was used to assess pharmacological similarity. We also combined each of these unique CDK inhibitors with venetoclax to assess interaction.

**Figure 4:**
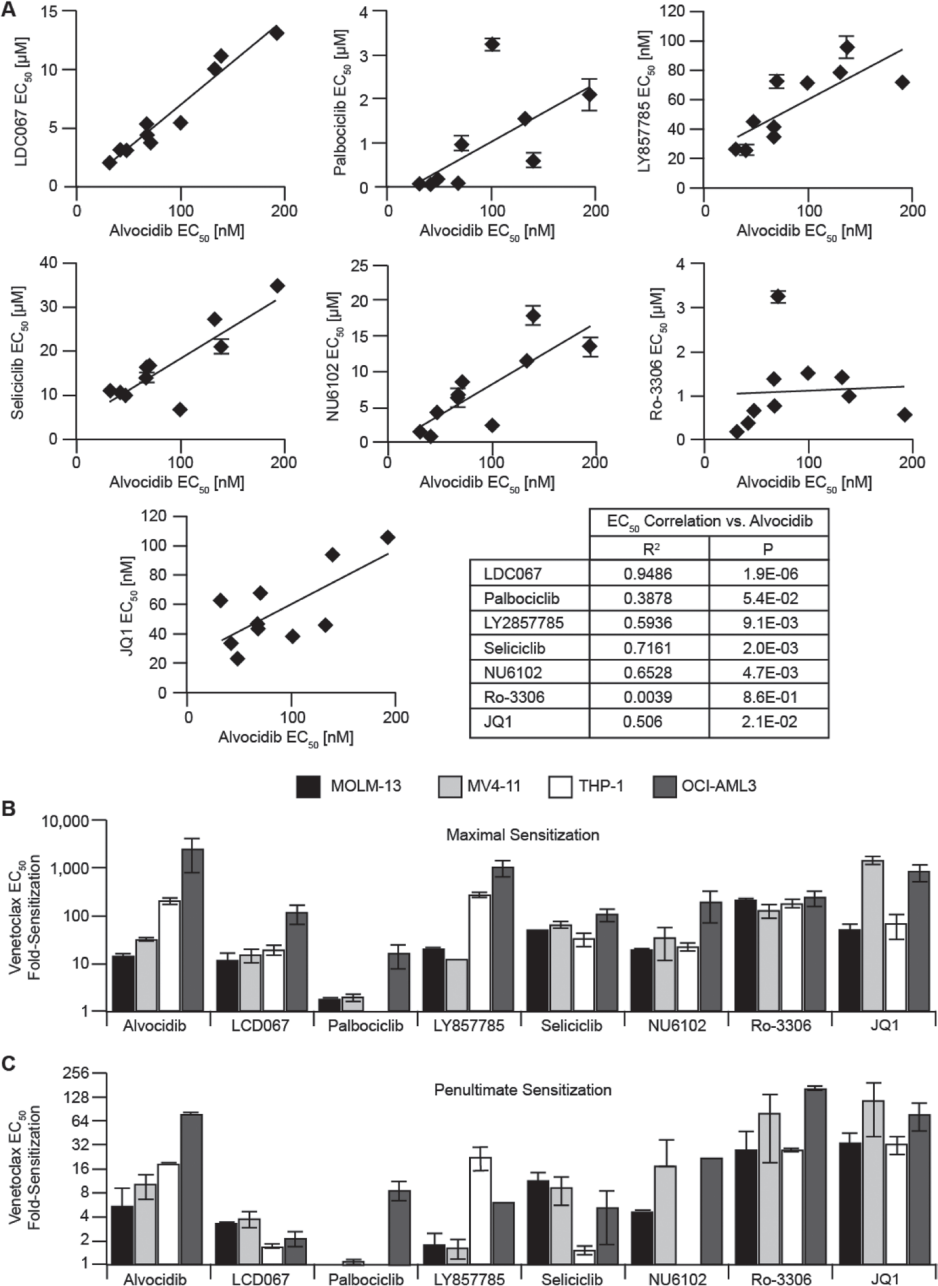
Pharmacological dissection of CDK isoforms contributing to venetoclax sensitization (**A**) single-agent drug dose response assays were assessed with duplicate biological experiments, each with four technical replicates, in a panel of eleven AML cell lines, and each EC_50_ value is plotted versus the EC_50_ value of alvocidib. Data represent average ± STD. Regression analysis was used to determine R^2^ and *P* values. (**B** and **C**), biological duplicate combination drug dose response assays, each with four technical replicates for every dose/dose combination evaluated, were performed with venetoclax and each inhibitor, and maximal venetoclax EC_50_ fold-sensitization (B) and penultimate EC_50_ fold-sensitization (**C**) plotted for each inhibitor. Data represent average ± STD.

In terms of single-agent EC_50_ correlation, CDK9-selective inhibitor LDC067 [37] showed the strongest correlation with alvocidib. Palbociclib, a recently FDA-approved CDK4/6-selective inhibitor, did not significantly correlate with alvocidib. Seliciclib (roscovitine), the most well-characterized CDK5 inhibitor which also inhibits CDK2, -7 and -9 with > 100-fold selectivity over CDK4 and -6 [38, 39], significantly correlated with alvocidib. LY2857785 [40], which compared to alvocidib more potently inhibits CDK8, shows less potency for CDK5 and similarly inhibits CDK9, correlated with alvocidib. NU6102, a CDK2 and -1 selective inhibitor [41] also significantly correlated with alvocidib. Ro-3306, a CDK1-selective inhibitor with 10-fold greater selectivity over CDK2 and 50-fold over CDK4 [42] (also reported to decrease BCL-2 protein in AML [43]) did not correlate with alvocidib. BRD4 inhibition with JQ1 exhibited a significant correlation with alvocidib. Notably, only alvocidib, LY857785, and JQ1 exhibited low nM EC_50_ values, while the remaining CDK inhibitors exhibited µM EC_50_ values (Figure 4A).

Regarding synergy with venetoclax, selective CDK9 inhibition with LDC067 only partially recapitulated the full magnitude of potentiation and dose-dependent activity of alvocidib. LDC067 was significantly less synergistic than alvocidib in venetoclax-resistant cells. Palbociclib showed marginal venetoclax fold-sensitization in venetoclax-sensitive cell lines and only at the highest (3 µM) dose. Further, palbociclib exhibited divergent activity in venetoclax-resistant cells, with dose-dependent antagonism observed in THP-1, and low-dose antagonism and high-dose sensitization observed in OCI-AML3. With seliciclib, venetoclax sensitization (ranging from 33- to 108-fold) was observed in all cells examined; however, venetoclax-sensitization occurred only at high (≥ 17.5 µM) seliciclib doses. Comparing equipotent doses, LY2857785 resulted in similar maximal venetoclax sensitization, although LY2857785 did not recapitulate the dose-dependent activity of alvocidib. NU6102 dose-dependently sensitized venetoclax in all cells examined, with maximal sensitization ranging from 20- to 193-fold at variable NU6102 doses from 2.5 to 20 µM. Despite poor single-agent correlation, Ro-3306 resulted in dose-dependent sensitization in all cells examined, with maximal venetoclax EC_50_ sensitization ranging from 130- to 242-fold. JQ1 generally recapitulated CDK inhibitor activity in potentiating venetoclax, exhibiting greater potentiation in venetoclax-sensitive lines (Figure 4B–4C and Supplementary Figure 3). We measured apoptosis to determine whether venetoclax potentiation with NU6102 or seliciclib was cytostatic or apoptotic. Similar to alvocidib, early and late apoptosis increased in the combination with each of these CDK inhibitors beyond either single-agent alone, albeit at higher µM doses (Supplementary Figure 4A–4B).

### Alvocidib synergizes with venetoclax in short-term *ex vivo* cultures of AML patient samples

**Figure 5:**
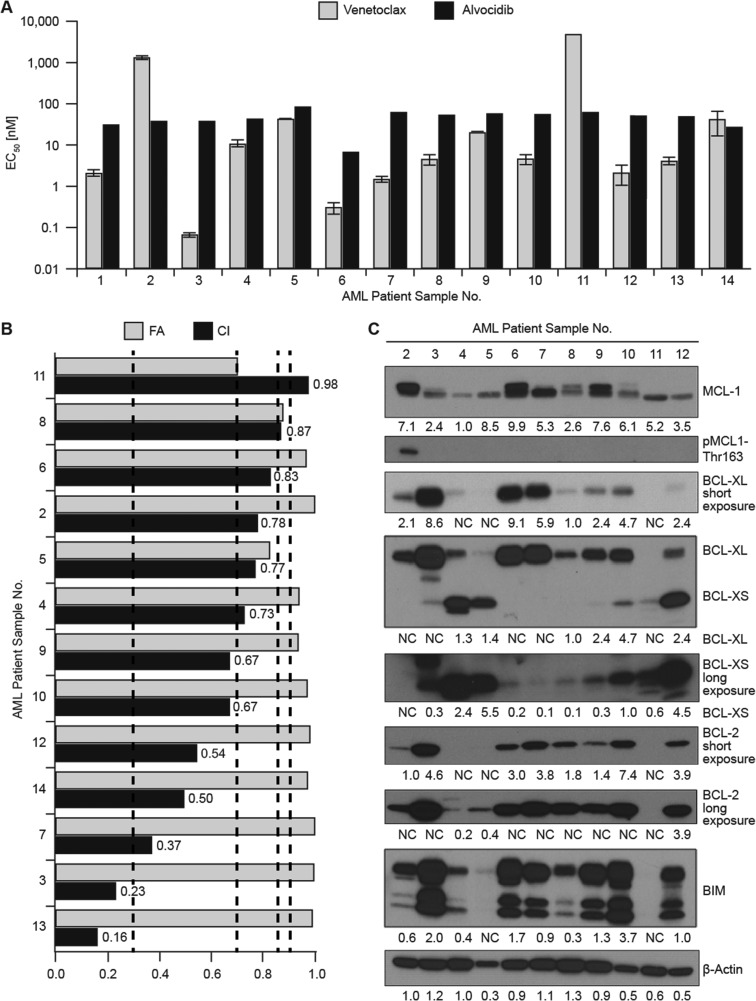

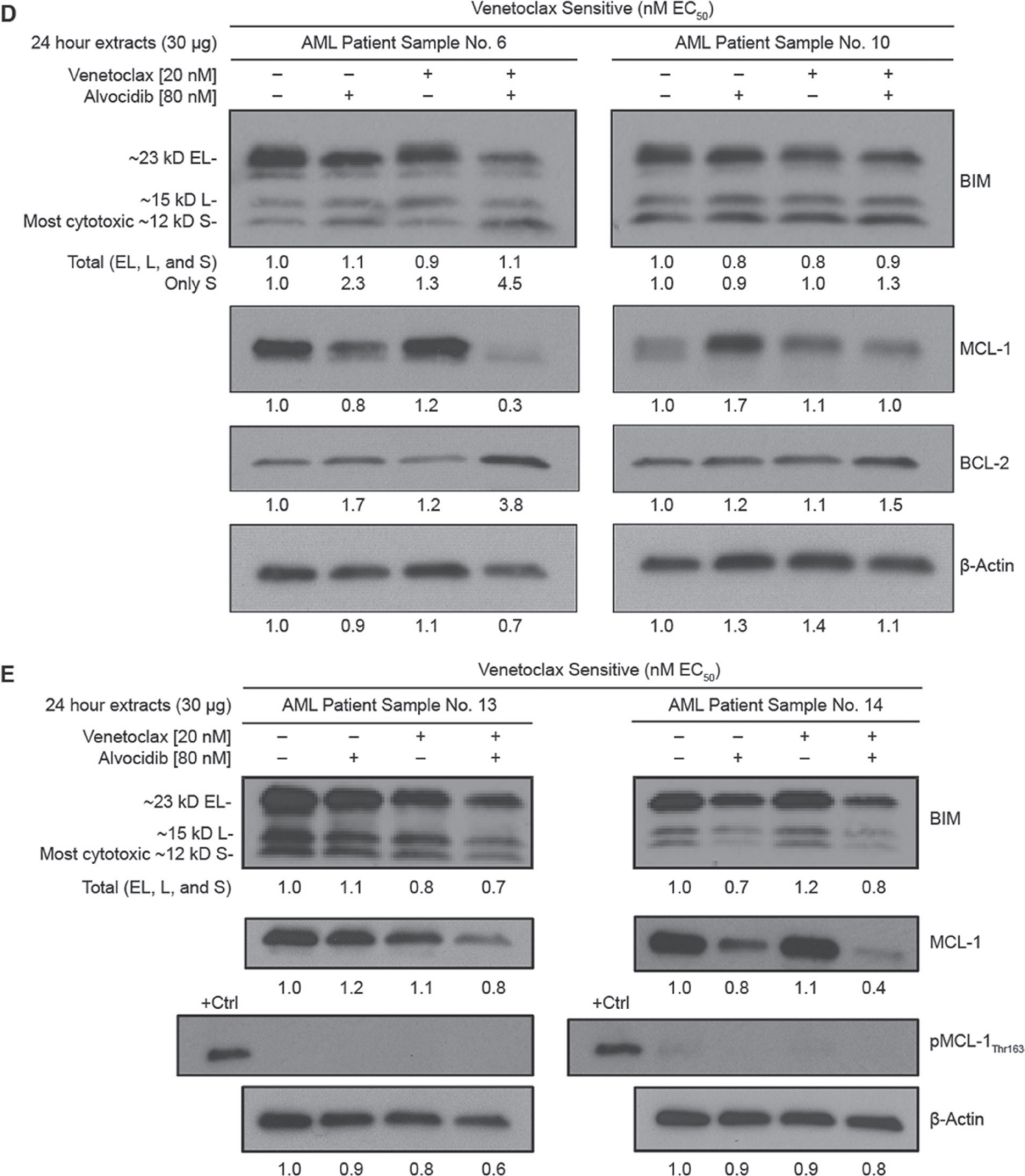
Alvocidib synergizes with venetoclax in short-term *ex vivo* cultures of AML patient samples and in a mouse model (**A**) twelve primary samples from bone aspirates of AML patients were treated with venetoclax and alvocidib in 96 hour culture, single-agent venetoclax and alvocidib EC50 values are plotted for each sample. Data represent average ± STD. (**B**) Combination Index (CI) values as an expression of synergy and Fraction Affected (FA) as an expression of fractional cell number (1.0 = maximal effect, while 0 = no effect) are shown for the combination of 40 nM venetoclax and 80 nM alvocidib as determined with CalcuSyn software. Dashed lines demark CI ranges: Strong synergy (< 0.3), synergy (0.3 to 0.7), moderate synergy (0.7 to 0.85), slight synergy (0.85 to 0.9), and additive (0.9 to 1.0). (**C**) untreated cell pellets were available for eleven of the fourteen primary samples analyzed in drug response assays, from which lysates were prepared for quantification of BCL-2 family proteins by western blot. (**D**) primary samples #6 and #10 after treatment with 20 nM venetoclax and 80 nM alvocidib, alone and in combination, for quantification of BIM, MCL-1 and BCL-2 by western blot. (**E**) primary samples #13 and #14 after treatment with 20 nM venetoclax and 80 nM alvocidib, alone and in combination, for quantification of BIM, MCL-1 and pMCL-1-Thr163 by western blot. For 5C–5E densitometry values calculated with Image J software are normalized to β-actin load controls. For *ex vivo* studies, primary sample material availability facilitated a single drug dose response assay for each sample, and a single lysate preparation for the indicated samples.

We analyzed combined venetoclax and alvocidib in patient samples cultured *ex vivo* (*N* = 14). Twelve of fourteen samples were confirmed as overt AML, one sample was derived from a patient with residual disease (sample #4), and one sample was derived from an AML patient in remission (sample #11). Of the samples analyzed, only one was resistant to venetoclax (sample #2; EC_50_ 1.3 ± 0.08 µM), while the remission sample was also resistant (EC_50_ > 5 µM) as expected. Median venetoclax EC_50_ was 5 nM (excluding the two resistant samples). Alvocidib single-agent activity was homogeneous (median EC_50_ 52 ± 19 nM), which for the remission sample was not unexpected as CDK inhibitors can cause cytopenias (Figure 5A). All of the confirmed AML samples exhibited synergy. Median maximal CI value was 0.52 ± 0.18, occurring at variable doses of venetoclax and alvocidib. For consistent comparison, we plotted CI values for median doses at which maximal synergy occurred (40 nM venetoclax, 80 nM alvocidib), alongside the fraction of cells affected, expressed as Fraction Affected (FA) (Figure 5B). At these low, clinically-achievable doses, 7 of 13 samples (54%) exhibited “synergy” as defined by CI values < 0.7, 4 of 13 (31%) exhibited “moderate synergy” defined by CI values of 0.7 to 0.85, while one sample had a CI of 0.87 defined as “slight synergy,” and remission sample #11 had a CI of 0.98 indicative of additive activity. The combination was synergistic in samples from patients clinically refractory to azacitidine (samples 7 & 13) (Supplementary Table 1).

We analyzed protein levels of selected BCL-2 family members in untreated extracts from eleven available samples used for *ex vivo* synergy assessment. Consistent with our previous report, BCL-2 family expression was overlapping within individual samples and heterogeneous across samples [14]. MCL-1 resolved as two distinct molecular weight (MW) bands, with some samples exhibiting both bands, while most samples predominantly expressed the lower MW band. MCL-1 phosphorylated at Thr163 was detectable only in the single *ex vivo* venetoclax-resistant sample (#2) (Figure 5C). BCL-XL, BCL-2 and BIM were not detected in the remission sample (#11). No individual BCL-2 family member correlated with synergy, as determined using CI values from Figure 5B (Supplementary Figure 5). Levels of BIM correlated most strongly with BCL-2, and also significantly correlated with BCL-XL, yet neither total MCL-1 nor high MW MCL-1 correlated with BIM, suggesting that this small sampling of primary AML samples was primarily dependent on BCL-2 followed by BCL-XL for survival (Supplementary Figure 6). For two samples (#6 and #10), sufficient sample quantity enabled treatments with venetoclax and/or alvocidib. Consistent with divergent *in vitro* results, we found that alvocidib decreased MCL-1 in sample #6, yet increased MCL-1 in sample #10. Total BIM was not significantly changed by alvocidib in either primary sample, while both showed increases in BCL-2 in the combination, consistent with *in vitro* results (Figure 5D). We identified and treated two additional AML samples to expand upon these results (presented in Figure 5A–5B as #13 and #14). Sample #14 exhibited a decrease in MCL-1 with 80 nM alvocidib, which was further decreased by the combination. Sample #13 showed a marginal decrease of MCL-1 only with the combination. Neither sample #13 or #14 showed increased BIM. MCL-1 Thr163 phosphorylation was undetectable or detected at only low levels, and was not significantly altered with any treatment (Figure 5E).

### Alvocidib potentiates venetoclax activity *in vivo*

**Figure 6:**
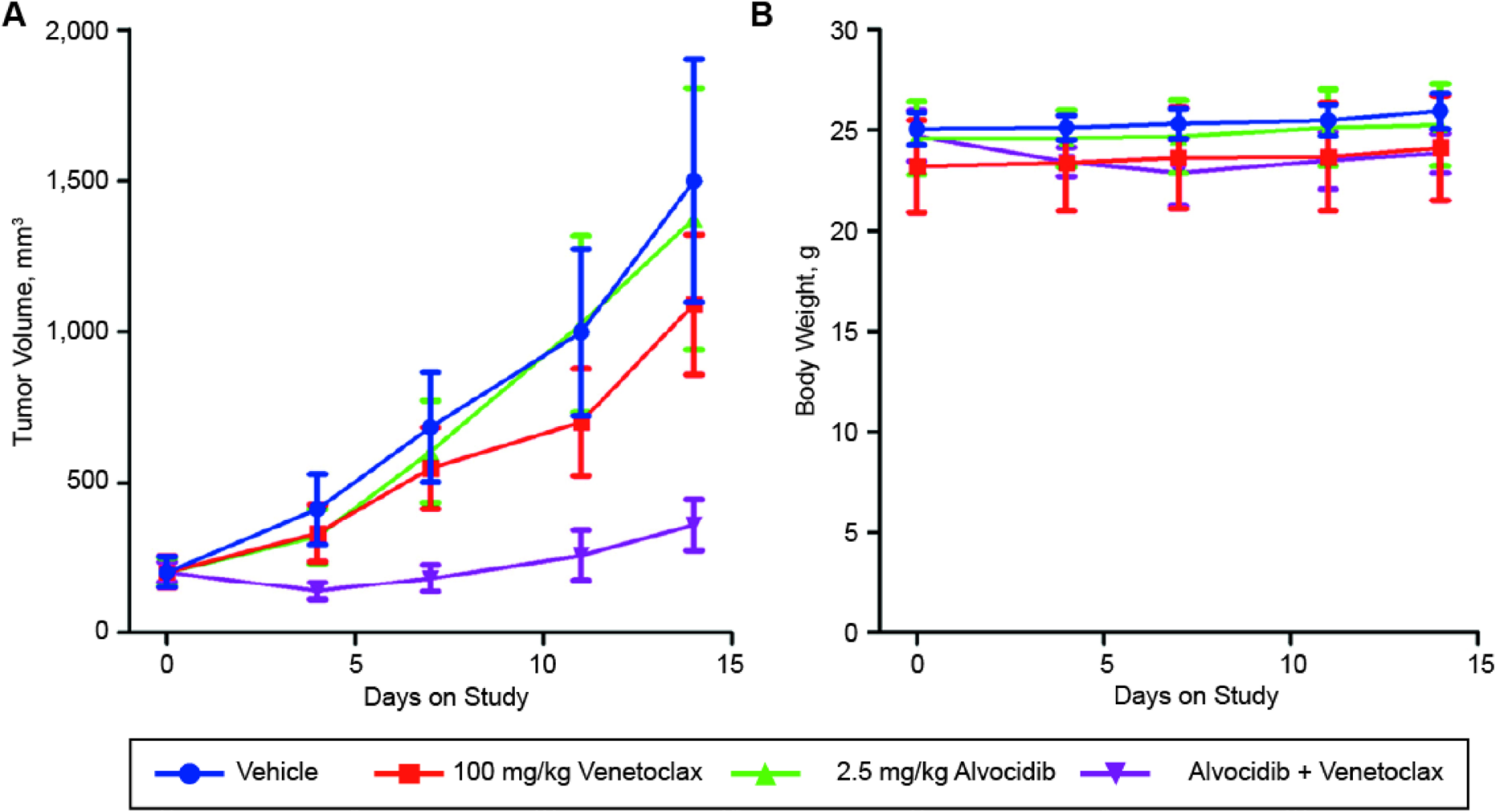
Alvocidib + venetoclax is an active regimen in the venetoclax-resistant AML xenograft (OCI-AML3) model Athymic nude mice were injected subcutaneously in the hind flank with OCI-AML3 cells. When tumors reached approximately 200 mm3, mice were randomized and then treated with vehicle, 100 mg/kg venetoclax, 2.5 mg/kg alvocidib, or a combination of the two drugs. Mice were dosed daily by oral gavage (venetoclax) or intraperitoneal injection (alvocidib). Tumor volumes (**A**) and body weights (**B**) were measured and recorded twice weekly. Tumor volumes and body weights shown are mean ± SEM of eight animals per treatment cohort.

To investigate alvocidib potentiation of venetoclax activity *in vivo*, we assessed venetoclax-resistant OCI-AML3 cells in a mouse xenograft model using tumor volume as the primary endpoint. Animal body weight was recorded throughout the study as a surrogate for treatment toxicity. Treatment of OCI-AML3 tumor-bearing mice with 100 mg/kg venetoclax resulted in a 31.6% average tumor growth inhibition (TGI) by day 14 relative to vehicle treated animals. Alvocidib treatment at 2.5 mg/kg resulted in a 9.7% TGI over the same period. However, the combination of alvocidib and venetoclax resulted in a significant reduction in tumor volume, corresponding to an 87.9% TGI at day 14 (Figure 6A). Neither venetoclax, nor alvocidib, resulted in a significant reduction in bodyweight. The combination was well-tolerated, with an average 7.5% reduction in bodyweight at day 7, comparable to that of venetoclax alone (Figure 6B).

## DISCUSSION

The synergistic anti-leukemic activity of combined venetoclax and alvocidib observed in venetoclax-sensitive and –resistant models of AML *ex vivo*, *in vivo*, and *in vitro* suggests that this combination has the potential to improve outcomes in patients regardless of sensitivity to single-agent venetoclax. To our knowledge, this is the first report exploring the potential synergy of venetoclax and alvocidib in AML. Considering the clinical relevance of alvocidib and venetoclax, as well as lack of effective treatment options for AML, we propose that the current report is distinct from prior reports evaluating BCL-2 inhibitors with CDK inhibitors, e.g. [44, 45].

In the current report, a decrease of anti-apoptotic MCL-1 was observed in most AML cells tested, while an increase of pro-apoptotic BH3-only proteins, predominantly BIM, was observed in other cell lines. MOLM-13, a cell line derived from an MDS patient transformed to AML, was unique amongst cell lines examined in that alvocidib did not decrease MCL-1, yet demonstrated strong synergy with venetoclax. Importantly, increases of all pro-apoptotic BH3-only proteins measured, including BIM, PUMA, NOXA and NBK, were observed in MOLM-13. This suggests that an increase of pro-apoptotic BH3-only proteins can be functionally equivalent to a decrease of anti-apoptotic MCL-1 with regard to synergy with venetoclax.

MicroRNAs from the polycistron miR-17–92, known to negatively regulate BIM [35, 36], were decreased in alvocidib-treated cells *in vitro*, although further experiments are needed to determine if this is a direct or indirect result of CDK9 inhibition. Combined venetoclax and alvocidib was found to increase pro-apoptotic BH3-only proteins beyond the increase observed with each single-agent for some cell lines. Thus, in addition to increased levels of “free” BH3-only protein(s) (i.e. unbound by anti-apoptotic proteins) resulting from BCL-2 inhibition by venetoclax, and/or alvocidib reduction of MCL-1 protein, alvocidib may have the capacity to induce apoptosis by increasing total BIM protein levels and/or other BH3-only proteins, although this activity was not observed in our small sampling of AML analyzed *ex vivo* (*n* = 4). MCL-1 was decreased by alvocidib and/or the combination in three of four AML patient samples assessed *ex vivo*, suggesting MCL-1 as a predominant mechanism in the primary cells analyzed.

Altogether, both *in vitro* and *ex vivo* results are consistent in showing divergent effects on BCL-2 family proteins despite similar levels of synergy, suggesting that no effect is absolutely associated with synergy per se, yet the overall balance of pro- and anti-apoptotic BCL-2 family proteins is consistently shifted in favor of apoptosis. Previous reports of exogenous manipulation of MCL-1, and dual targeting of MCL-1 and BCL-2, support this concept as it relates to alvocidib and enhanced venetoclax activity [26, 46–48]. Importantly, abrogation of synergy with either BIM knock-down or BCL-XL over-expression also supports this concept and provides strong functional evidence that the majority of synergy between venetoclax and alvocidib is based on mechanism(s) that converge upon the intrinsic apoptotic pathway. More specifically, while BIM knock-down showed a trend towards abrogating single-agent venetoclax activity, the change was not significant, suggesting that, at least in the two AML cell lines analyzed, single-agent venetoclax operates largely independent of BIM through the classic displacement model [49], whereby BCL-2 directly interacts with BAX/BAK to negatively regulate MOMP. Conversely, BIM knock-down abrogated the majority of synergy between venetoclax and alvocidib, suggesting that the combination is at least partially dependent on the direct activation model [49], whereby BIM acts as sensitizer and/or activator of BAX/BAK. To our knowledge the effect of BIM knock-down has not previously been examined in the context of the proposed combination, thus abrogation of synergy by BIM knock-down is an important insight into the mechanism of apoptotic synergy between alvocidib and venetoclax. Importantly, reduction of MCL-1 by alvocidib similarly has the capacity to operate through the displacement model, by decreasing MCL-1 available to inhibit BAX/BAK, as well as the direct activation model, by decreasing MCL-1 available to act as a reservoir for sequestration of BIM and/or other BH3-only proteins.

Using multiple complementary approaches, we clearly demonstrate that BCL-XL counteracts venetoclax/alvocidib synergy *in vitro*. Results with BCL-XL^High^ cells are consistent with a model whereby BCL-XL acts in parallel to BCL-2 to inhibit BAX/BAK and/or sequester BIM/BH3-only proteins, and with a model whereby BCL-XL can also counteract MCL-1 reduction and/or BH3-only protein increases by alvocidib. Given the totality of *in vitro* data regarding BCL-XL, we were surprised to observe increased BCL-XL protein in alvocidib treated samples *in vitro*. Importantly however, the levels of increased BCL-XL observed in BCL-XL^Low^ cells were still strikingly lower than baseline levels observed in BCL-XL^High^ cells, demonstrating that absolute levels of BCL-XL may be an important determinant of response *in vitro*. BCL-XL levels did not correlate with venetoclax single-agent activity or synergy with alvocidib in our small sampling of AML patients *ex vivo*, suggesting that BCL-XL may not represent a major limitation to clinical efficacy of the combination in AML. However, several questions regarding BCL-XL as an *in vivo* mechanism of resistance to the combination remain unanswered, such as differences in relative versus absolute levels of BCL-XL *in vitro* versus *in vivo*, as well as potential cell lineage effects of BCL-XL function [50]. It remains to be determined clinically whether some cases of AML with high BCL-XL expression (putatively erythroleukemia or megakaryoblastic lineage expression of BCL-XL) may be more resistant to the proposed combination of venetoclax and alvocidib, and thus benefit more from dual BCL-2 and BCL-XL inhibition (i.e. navitoclax/ABT-737/ABT-263) alone or combined with alvocidib. Nonetheless, our observations are consistent with recent reports showing that mechanisms of venetoclax resistance are not universal or mutually exclusive [51, 52].

Upon examination of BCL-2 family proteins after combined venetoclax and alvocidib treatment, we were surprised to observe increased MCL-1, BCL-2 and BCL-XL. However, we observed a corresponding increase in BIM beyond the increase attributable to single-agent alvocidib in most cells, while NOXA was observed to be increased in the combination, beyond either single-agent, in the only cell line for which we did not observe strong BIM up-regulation at the same time point. BIM and BCL-2 have the capacity to mutually regulate each other, thus we speculate that the increase in anti-apoptotic BCL-2 family proteins could be a response to increased BIM, or vice versa [53]. Nonetheless, the data presented herein is consistent with the hypothesis that the net balance of pro- and anti-apoptotic BCL2 family members determines intrinsic apoptotic response in general, as well as for the proposed combination. BH3 profiling, an evolving assay that functionally probes the intrinsic apoptotic pathway, could therefore prove to be a useful predictive biomarker for this regimen [4, 14, 44, 54, 55].

We found that CDK9-selective inhibition correlated strongly with venetoclax potentiation. While the magnitude of venetoclax sensitization with LDC067 was significantly lower, especially in venetoclax-resistant cells, a strong correlation was observed. We speculate these distinctions are due to differences in potency for CDK9, although inhibition of other CDK isoforms cannot be ruled-out. We did not test the CDK9 inhibitor dinaciclib in this study, as previous studies in diffuse large B-cell lymphoma (DLBCL) have been reported [56]. While a stronger single-agent potency of dinaciclib versus alvocidib in DLBCL cell lines was reported, a clear and comparable potentiation venetoclax upon combined dinaciclib was also reported, regardless of the single-agent potency of either dinaciclib or venetoclax in the DLBCL cell lines used. These observations are consistent with our findings demonstrating robust synergy regardless of venetoclax single-agent activity in AML. CDK4/6 inhibition with palbociclib did not generally potentiate venetoclax activity, and was actually antagonistic in some AML cells. While we cannot rule-out the possibility that CDK4/6 inhibition contributes to venetoclax sensitization under concurrent CDK9 inhibition, these results suggest that palbociclib may not be an ideal candidate for combination with venetoclax in AML. Strong venetoclax potentiation was also observed with seliciclib, NU6102, and Ro-3306; however, while these CDK inhibitors do not potently inhibit CDK9, they likely inhibit CDK9 at the relatively high doses required to elicit the synergy observed. In comparing equipotent doses of LY2857785 or alvocidib combined with venetoclax, maximal venetoclax fold-sensitization was similar for both compounds, although synergy at the next closest dose was lower for LY2857785, especially for venetoclax -resistant cells. BRD4 inhibitor JQ1 resulted in dose-dependent venetoclax sensitization similar to alvocidib, suggesting that inhibition of P-TEFb through BRD4 is functionally similar to CDK9 inhibition. As JQ1 unlikely has off-target effects on CDKs, this indirect evidence supports the notion that alvocidib predominantly synergizes with venetoclax through CDK9 inhibition.

The functional and mechanistic evidence presented herein provide strong pre-clinical precedence for clinically investigating the efficacy of combined alvocidib and venetoclax in AML. Clinical studies have shown that alvocidib can be safely added to induction therapy with acceptable toxicity [57], thus the combination may be clinically feasible. However, the cytopenias observed with alvocidib, and recently with combined venetoclax and azacitidine (including unexpected thrombocytopenia), emphasize the importance of using the lowest possible doses, and highlights the importance of low dose synergy we consistently demonstrate herein. Development of a clinical trial testing combined alvocidib and venetoclax in AML and high-risk MDS is ongoing.

## MATERIALS AND METHODS

### Cells, culture conditions and reagents

Primary samples were obtained with informed consent in accordance with Mayo Clinic IRB-approved research protocols and handled according to Good Clinical Practice. Primary cells were Ficoll-gradient separated, and immediately cultured, or viably frozen for subsequent short-term culture. Cell lines were obtained from ATCC or DSMZ. All cell lines tested negative for mycoplasma before cryopreservation, and all cell lines were confirmed free of cross-contamination using PCR-based DNA fingerprinting. Cells were cultured in RPMI-1640 containing 10% FBS, 2 mM L-glutamine, 100 IU/mL penicillin, 100 µg/mL streptomycin (Invitrogen) at 37°C/5% CO2. Compounds were obtained as follows: Alvocidib (Tolero Pharmaceuticals), venetoclax/ABT-199 (ChemieTek), ABT-737, LDC067, palbociclib, seliciclib and Ro-3306 (SelleckChem), LY2857785 (MedChem Express), NU6102 and JQ1 (Cayman Chemical).

### Drug dose response assays and CalcuSyn analysis

Cells were plated in 384-well plates (Greiner Bio-One) at 1000 cells/well for cell lines, or 2000 cells/well for primary samples, and dosed simultaneously for combinations. Relative cell number (expressed as % viability) was measured with CellTiter-Glo (Promega) at 96 hours using a Cytation3 plate reader (BioTek). Nine doses of venetoclax titrated specifically for venetoclax-resistant or -sensitive cells, or ABT-737, were combined with six doses of the second drug evaluated, yielding 54 possible combinations, each evaluated in quadruplicate for every experiment. For primary cells, where venetoclax sensitivity was unknown a priori, venetoclax was serially diluted 5-fold (5.0 µM to 0.013 nM), which yielded at least three doses for CalcuSyn analysis, version 2.1 (Biosoft) [58, 59]. Prism version 5.03 (Prism Software Corporation) was used to calculate EC_50_ values.

### Flow cytometry

Cells were seeded at 2E5 cells/mL and incubated 24 hours with appropriate CDK inhibitor (80 nM alvocidib, 17.5 µM seliciclib, or 2.5 or 20 µM NU6102) with or without 2.5 or 10 nM venetoclax for venetoclax-sensitive cells, or 0.25 or 1 µM venetoclax for venetoclax -resistant cells. Cells were washed with ice-cold PBS, suspended in binding buffer at 1E6 cells/mL, Annexin V antibody added at [1:20] (BD Biosciences) and propidium iodide (Sigma Aldrich) added to [5 µg/mL]. After incubating 15 minutes, cells were analyzed on LSR Fortessa (BD Biosciences).

### Western blots

After treatment with alvocidib and/or venetoclax at indicated doses/times, protein was harvested with lysis buffer (Cell Signaling; #9803) containing 1 mM PMSF. Lysates were quantified by BCA (Pierce/Thermo Fisher), 26–30 µg total protein resolved by 4–15% SDS-PAGE, transferred to PVDF membranes (80V/90 minute wet-transfer), and blocked with 5% non-fat dry milk before primary antibody incubation, 4°C/overnight. MCL-1 (#4527), pMCL-1-Thr163 (#1476), BCL-2 (#2872), BCL-XL (#2762), BIM (#2819), PUMA (#4976), β-tubulin (#2128) (Cell Signaling), NBK (sc-365625; Santa Cruz Biotechnology), NOXA (ab140129; Abcam), and β-actin (A00702; GenScript). Densitometry was performed with Image J software (www.imagej.nih.gov).

### Lentivirus construction

BCL-XL (cDNA clone ID 2823498, GE Dharmacon) was amplified by PCR and cloned into plasmid pSC11CMVFlag, with a CMV promoter and 5′3Xflag tag. BCL-XL was then cloned into lentiviral vector pWPI (Addgene plasmid #12254) resulting in plasmid pWPIS6FBCL-XL. Plasmid pWPIS6FBCL-XL, together with packaging plasmid psPAX2 (plasmid #12660) and pMG2.g (plasmid #12259) were transfected into 293T cells and rescued into lentivirus.

### Quantitative RT-PCR

MicroRNAs were isolated using Directzol RNA mini-prep kit (Zymo Research). Reverse transcription was performed using TaqMan MicroRNA RT Kit, resulting cDNA underwent pre-amplification with TaqMan PreAmp Master Mix, and RTqPCR was performed with TaqMan MicroRNA assays and TaqMan Universal Master Mix II containing UNG (Life Technologies).

### Small-interfering RNA transfection

siRNA were purchased from Qiagen. For target protein knock-down validation, 0.04 nmol siRNA was mixed with 750 µL RNAiMax transfection reagent (ThermoFisher) diluted in OPTI-MEM (Invitrogen) (dilutions optimized per cell line and reagent batch), after 30 minutes 2.6 to 4.3E5 cells (optimized per cell line) were added in 1 mL unsupplemented RPMI-1640, spiked with FBS to [2.2%], and harvested at 48 hours. For siRNA drug dose response assays, siRNA were pre-printed on 384-well plates, OPTI-MEM-diluted transfection reagent was added at 20 µL/well, after 30 minutes 1000–2000 cells/well were added in 20 µL of unsupplemented RPMI-1640, and spiked with FBS to [2.2%]. For BIM siDDRs, plates were incubated for 24 hours prior to simultaneous dosing of venetoclax and alvocidib and read 48 hours later. For BCL-XL siDDRs, plates were incubated for 48 hours prior to simultaneous addition of venetoclax or ABT-737 and alvocidib and read 48 hours later.

### Mouse studies

Animal studies were reviewed and approved for ethical consideration by an internal review committee at Tolero Pharmaceuticals, Inc. OCI-AML3 cells were transplanted into 6–8 week old, female, hairless outbred SCID mice (Crl:SHO-Prkdc^scid^Hr^hr^) (18–26 g, strain code 474, Charles River Laboratories). Study animals were housed under standard conditions, and given food and water ad libitum. 1E7 OCI-AML3 cells were suspended in serum-free media, mixed 1:1 in matrigel (Corning), and injected subcutaneously into the hind flank. Randomization and treatment was initiated once tumor volumes had reached approximately 200 mm^3^. Alvocidib was formulated in saline, while venetoclax was formulated in Phosal 50PG: PEG400: Ethanol. Mice were administered vehicle or drug treatment by intraperitoneal injection, (qdx5)x3 with alvocidib (2.5 mg/kg), by oral gavage (qdx5)x3 with venetoclax (100 mg/kg), or both alvocidib and venetoclax. Tumor length and width measurements were made with digital calipers, and tumor volumes were calculated as follows: length x width x width/2, where length was the longest diameter. Tumor volume and body weights were measured and recorded twice weekly.

### Statistics

*P* values were calculated using two-tailed Student’s *t*-test. For correlative comparisons of drug EC_50_ values, or drug activity with protein level, regression analysis was used to calculate R^2^ and corresponding *p* values. R values shown in Figure 1B were calculated with CalcuSyn software.

## SUPPLEMENTARY MATERIALS FIGURES AND TABLE


