# Supplementary material for: Combined venetoclax and alvocidib in acute myeloid leukemia
Source: Oncotarget. 2017 Nov 3;8(63):107206–22. doi: 10.18632/oncotarget.22284 (PMC5739808; doi:10.18632/oncotarget.22284)
Supplement: Supplementary file 1 [file oncotarget-08-107206-s001.pdf]

Combined venetoclax and alvocidib in acute myeloid leukemia

SUPPLEMENTARY MATERIALS

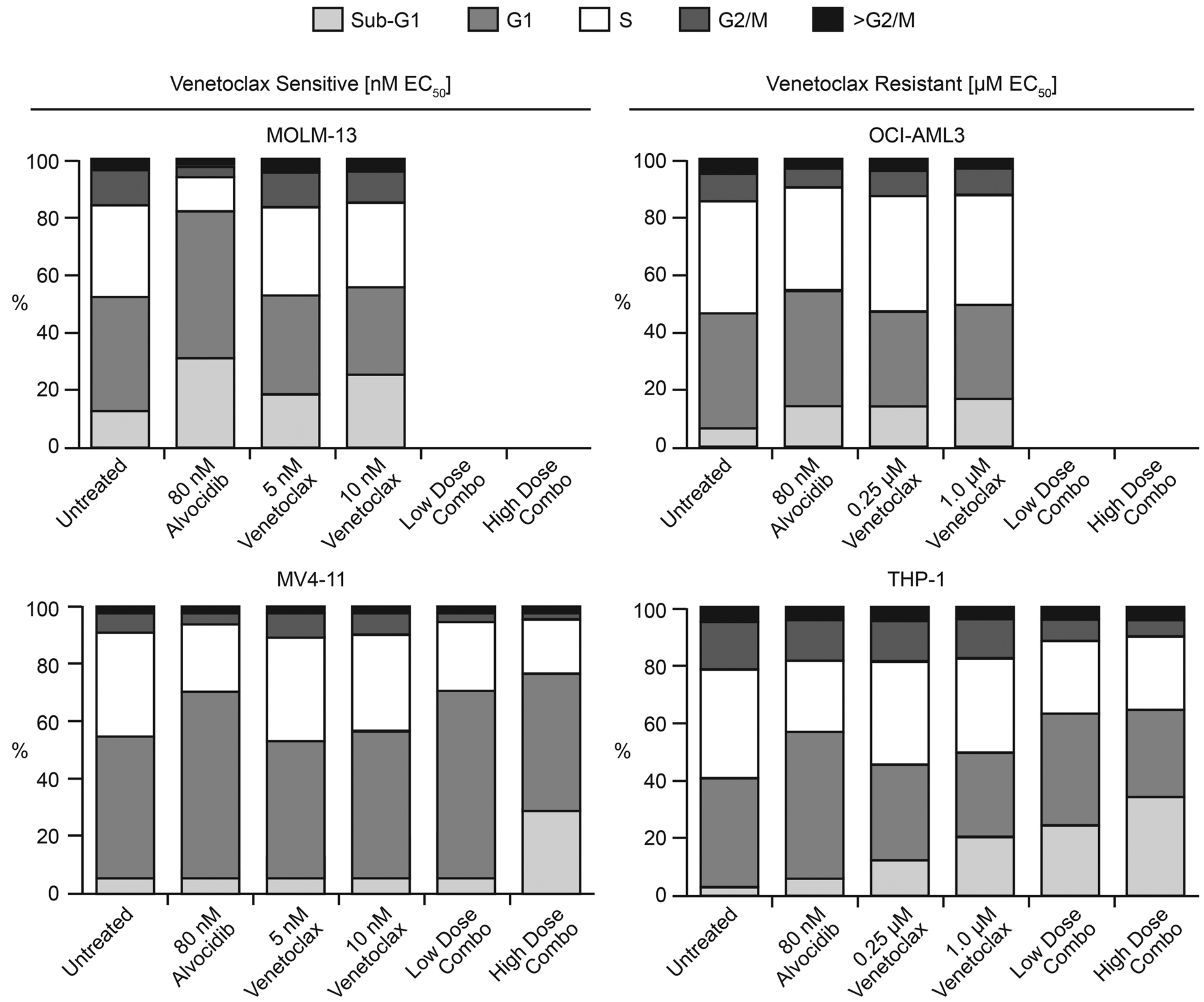

Supplementary Figure 1: Cell cycle analysis shows 80 nM alvocidib results in only moderate changes to cell cycle distributions. DNA content was determined using propidium iodide staining after the 24 hour treatment indicated on the x-axis, and cell cycle analysis performed with FlowJo software and manually defining G1 and G2 constraint ranges. Venetoclax-sensitive cells are shown in the left panel, and Venetoclax-resistant cells in the right panel.

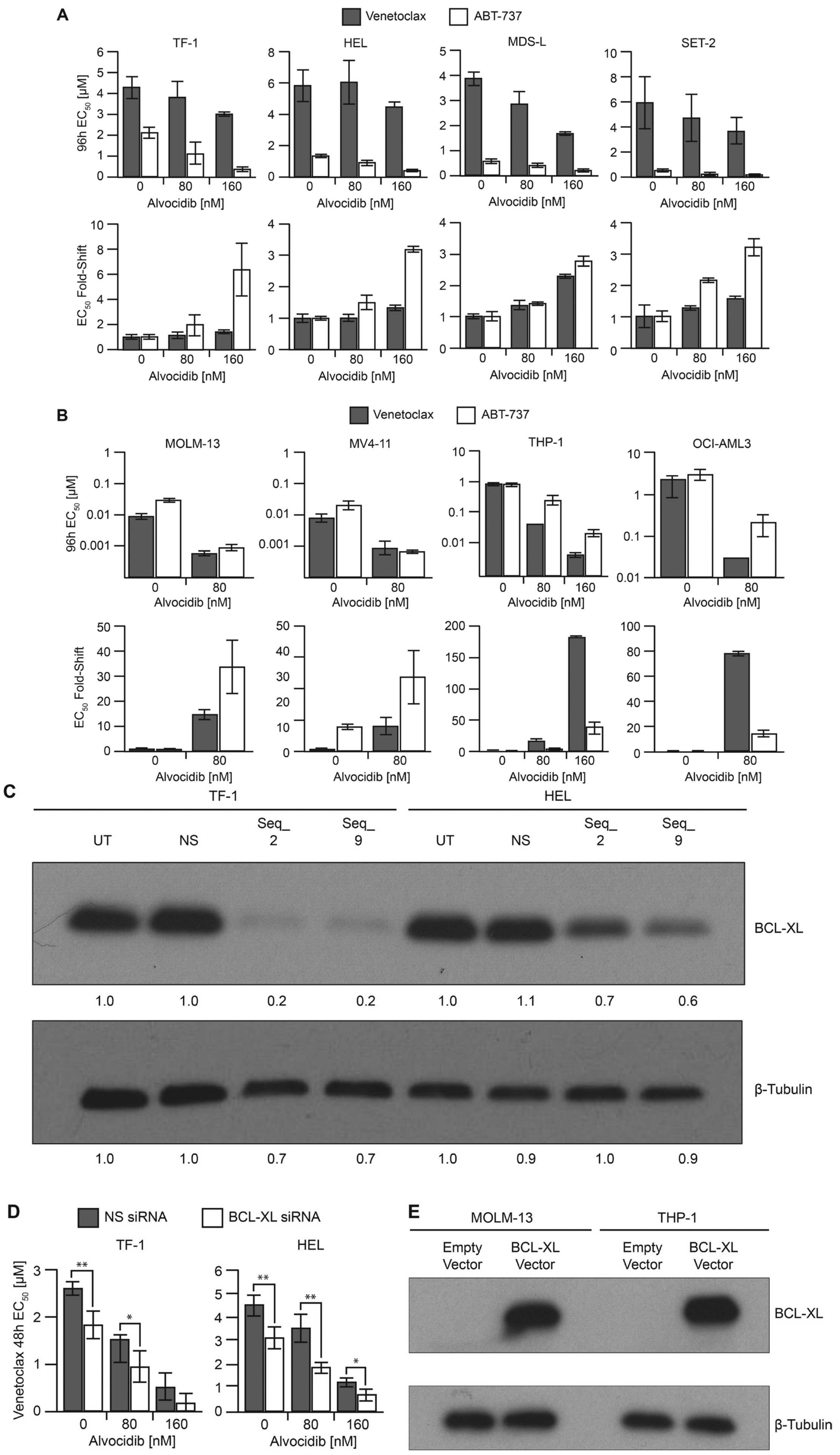
A
Venetoclax
ABT-737
TF-1
HEL
MDS-L
SET-2
96h EC50 [μM]
EC50 Fold-Shift
Alvocidib [nM]
B
Venetoclax
ABT-737
MOLM-13
MV4-11
THP-1
OCI-AML3
96h EC50 [μM]
EC50 Fold-Shift
Alvocidib [nM]
C
TF-1
HEL
UT
NS
Seq_2
Seq_9
BCL-XL
1.0 1.0 0.2 0.2 1.0 1.1 0.7 0.6
β-Tubulin
1.0 1.0 0.7 0.7 1.0 0.9 1.0 0.9
D
NS siRNA
BCL-XL siRNA
TF-1
HEL
Venetoclax 48h EC50 [μM]
Alvocidib [nM]
E
MOLM-13
THP-1
Empty Vector
BCL-XL Vector
BCL-XL
β-Tubulin

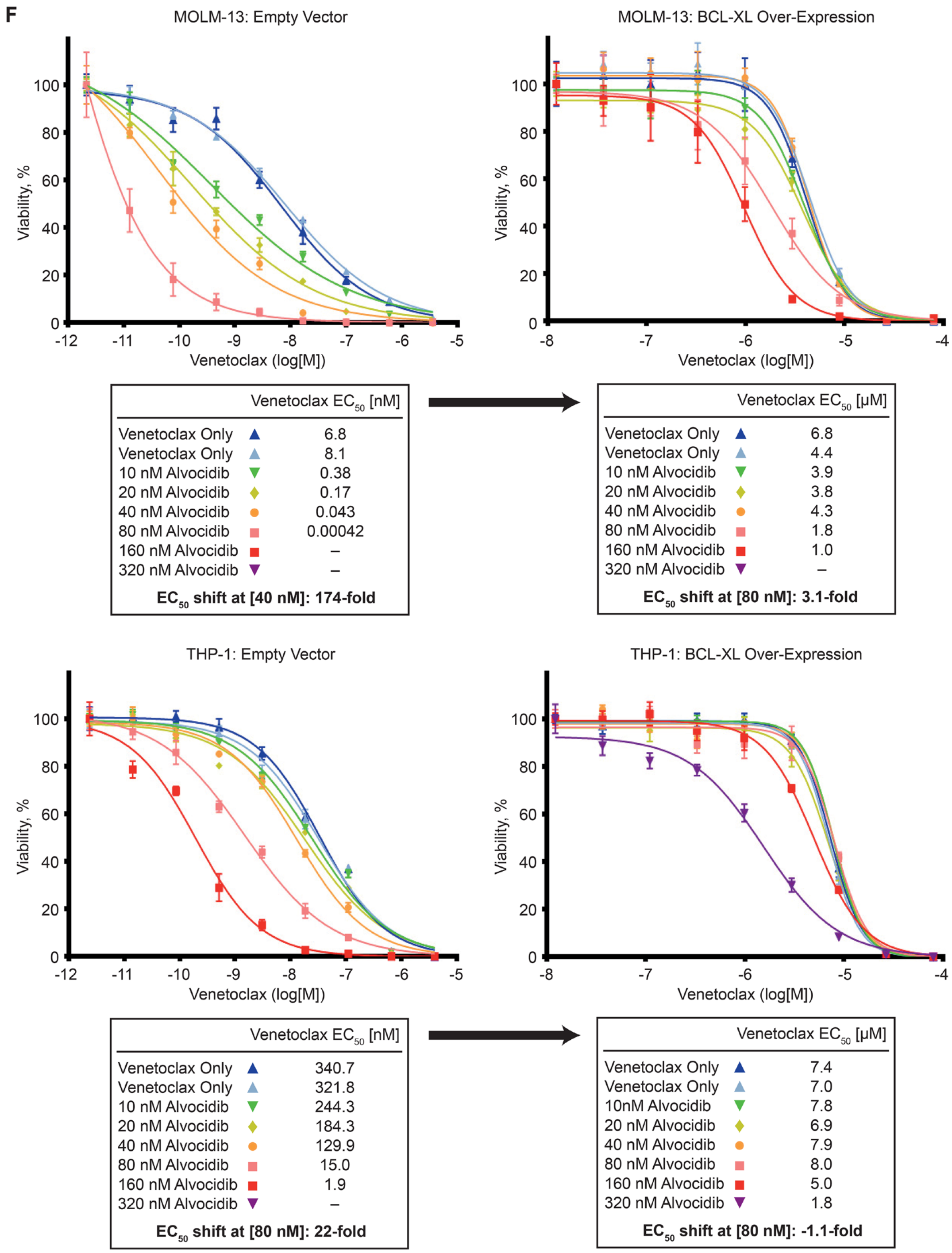

Supplementary Figure 2: BCL-XL modulates venetoclax response and alvocidib potentiation of venetoclax in AML models in vitro. AML cell lines with high BCL-XL expression (TF-1, HEL, MDS-L and SET-2) (**A**), and low BCL-XL expression (MOLM13, MV4-11, THP-1 and OCI-AML3) (**B**) were assessed in duplicate biological drug dose response experiments comparing venetoclax versusABT-737 +/- alvocidib. Venetoclax EC_{50} values are plotted versus ABT-737 EC_{50} values with increasing concentrations of alvocidib(upper panel A, B), while corresponding venetoclax or ABT-737 EC_{50} fold-sensitization is plotted in the lower panel of A and B.C, TF-1 and HEL lysates prepared after treatment with non-silencing (NS) or BCL-XL siRNA were resolved by gel electrophoresis to quantify specific BCL-XL knock-down. (**D**), siRNA targeting BCL-XL were compared against NS siRNA in cells treated with thecombination of venetoclax and alvocidib, and EC_{50} values plotted with increasing concentrations of alvocidib (data from BCL-XLsiRNA sequence 2 and sequence 9 were compiled from duplicate biological experiments each assessing four technical replicatesfor each sequence and for every dose evaluated). *P* values were calculated using a two-tailed Student's *T*-test. $^{*}P$ value < 0.05,$^{**}P$ value < 0.02. BCL-XL was over-expressed (**E**) with a lentiviral construct in AML cell lines with low endogenous BCL-XL expression and treatment with venetoclax and/or alvocidib was assessed in comparison to empty vector control virus (F). For A-B & D, data represent average ± STD.

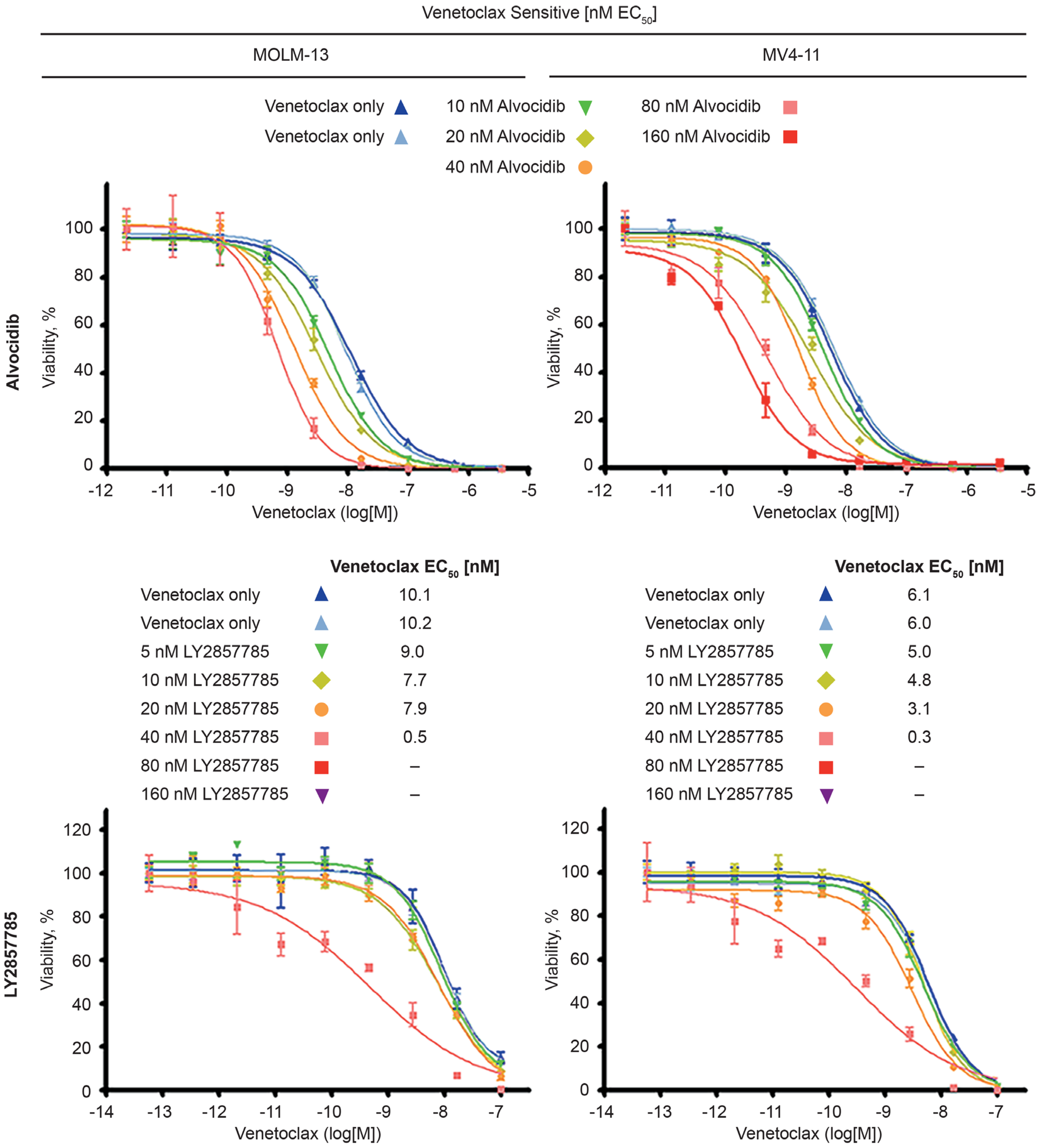
Venetoclax Sensitive [nM EC50]
MOLM-13
MV4-11
Venetoclax only
Venetoclax only
10 nM Alvocidib
20 nM Alvocidib
40 nM Alvocidib
80 nM Alvocidib
160 nM Alvocidib
Alvocidib
Viability, %
Venetoclax (log[M])
Venetoclax EC50 [nM]
Venetoclax only 10.1
Venetoclax only 10.2
5 nM LY2857785 9.0
10 nM LY2857785 7.7
20 nM LY2857785 7.9
40 nM LY2857785 0.5
80 nM LY2857785 –
160 nM LY2857785 –
Venetoclax EC50 [nM]
Venetoclax only 6.1
Venetoclax only 6.0
5 nM LY2857785 5.0
10 nM LY2857785 4.8
20 nM LY2857785 3.1
40 nM LY2857785 0.3
80 nM LY2857785 –
160 nM LY2857785 –
LY2857785
Viability, %
Venetoclax (log[M])

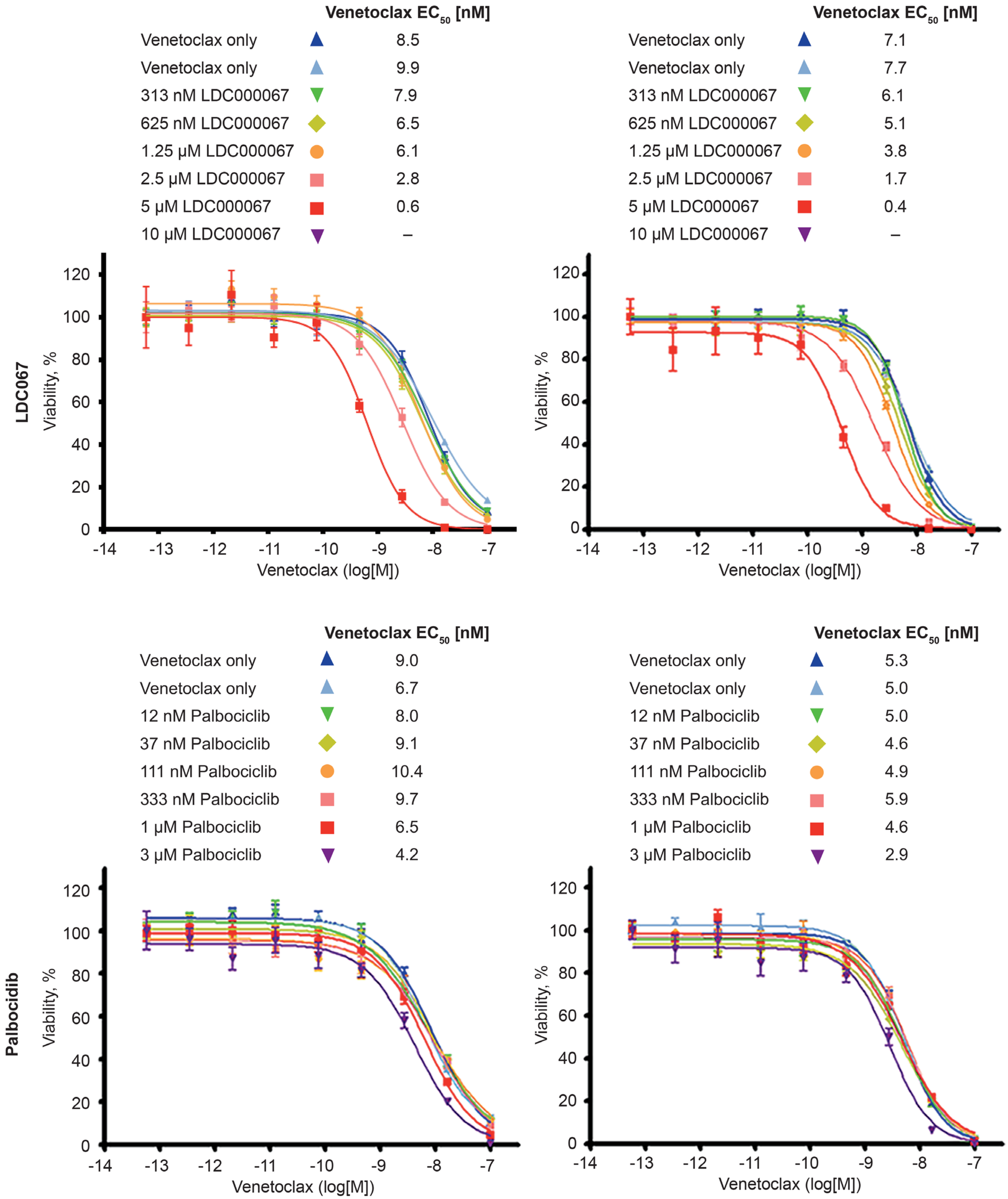
Venetoclax EC_{50} [nM]
Venetoclax only 8.5
Venetoclax only 9.9
313 nM LDC000067 7.9
625 nM LDC000067 6.5
1.25 µM LDC000067 6.1
2.5 µM LDC000067 2.8
5 µM LDC000067 0.6
10 µM LDC000067 –
LDC067
Viability, %
Venetoclax (log[M])
Venetoclax EC_{50} [nM]
Venetoclax only 7.1
Venetoclax only 7.7
313 nM LDC000067 6.1
625 nM LDC000067 5.1
1.25 µM LDC000067 3.8
2.5 µM LDC000067 1.7
5 µM LDC000067 0.4
10 µM LDC000067 –
Viability, %
Venetoclax (log[M])
Venetoclax EC_{50} [nM]
Venetoclax only 9.0
Venetoclax only 6.7
12 nM Palbociclib 8.0
37 nM Palbociclib 9.1
111 nM Palbociclib 10.4
333 nM Palbociclib 9.7
1 µM Palbociclib 6.5
3 µM Palbociclib 4.2
Palbocidib
Viability, %
Venetoclax (log[M])
Venetoclax EC_{50} [nM]
Venetoclax only 5.3
Venetoclax only 5.0
12 nM Palbociclib 5.0
37 nM Palbociclib 4.6
111 nM Palbociclib 4.9
333 nM Palbociclib 5.9
1 µM Palbociclib 4.6
3 µM Palbociclib 2.9
Viability, %
Venetoclax (log[M])

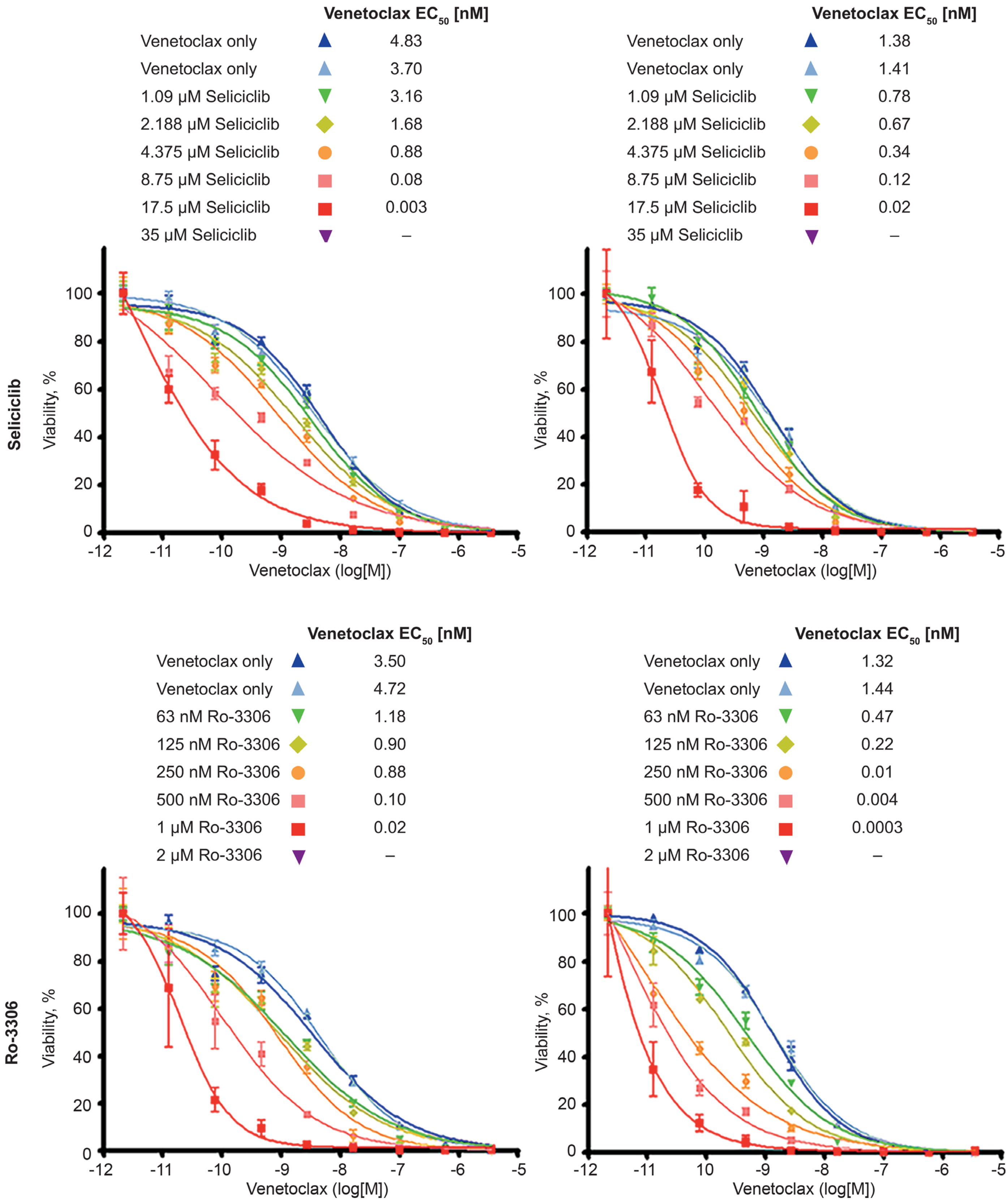
Venetoclax EC50 [nM]
Venetoclax only 4.83
Venetoclax only 3.70
1.09 μM Seliciclib 3.16
2.188 μM Seliciclib 1.68
4.375 μM Seliciclib 0.88
8.75 μM Seliciclib 0.08
17.5 μM Seliciclib 0.003
35 μM Seliciclib –
Venetoclax EC50 [nM]
Venetoclax only 1.38
Venetoclax only 1.41
1.09 μM Seliciclib 0.78
2.188 μM Seliciclib 0.67
4.375 μM Seliciclib 0.34
8.75 μM Seliciclib 0.12
17.5 μM Seliciclib 0.02
35 μM Seliciclib –
Seliciclib
Viability, %
Venetoclax (log[M])
Venetoclax EC50 [nM]
Venetoclax only 3.50
Venetoclax only 4.72
63 nM Ro-3306 1.18
125 nM Ro-3306 0.90
250 nM Ro-3306 0.88
500 nM Ro-3306 0.10
1 μM Ro-3306 0.02
2 μM Ro-3306 –
Venetoclax EC50 [nM]
Venetoclax only 1.32
Venetoclax only 1.44
63 nM Ro-3306 0.47
125 nM Ro-3306 0.22
250 nM Ro-3306 0.01
500 nM Ro-3306 0.004
1 μM Ro-3306 0.0003
2 μM Ro-3306 –
Ro-3306
Viability, %
Venetoclax (log[M])

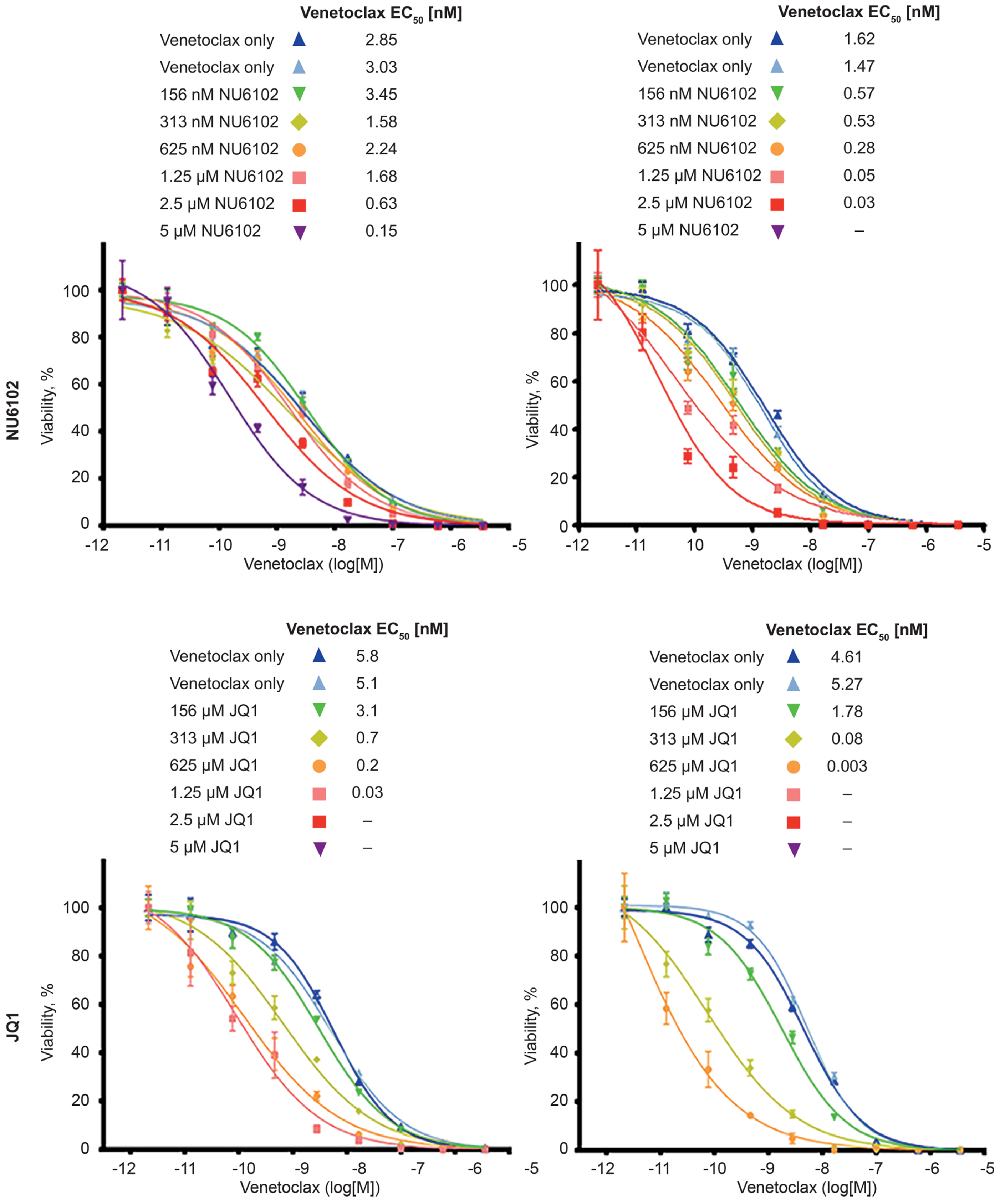
Venetoclax EC50 [nM]
Venetoclax only 2.85
Venetoclax only 3.03
156 nM NU6102 3.45
313 nM NU6102 1.58
625 nM NU6102 2.24
1.25 µM NU6102 1.68
2.5 µM NU6102 0.63
5 µM NU6102 0.15
Venetoclax EC50 [nM]
Venetoclax only 1.62
Venetoclax only 1.47
156 nM NU6102 0.57
313 nM NU6102 0.53
625 nM NU6102 0.28
1.25 µM NU6102 0.05
2.5 µM NU6102 0.03
5 µM NU6102 –
NU6102
Viability, %
Venetoclax (log[M])
Venetoclax EC50 [nM]
Venetoclax only 5.8
Venetoclax only 5.1
156 µM JQ1 3.1
313 µM JQ1 0.7
625 µM JQ1 0.2
1.25 µM JQ1 0.03
2.5 µM JQ1 –
5 µM JQ1 –
Venetoclax EC50 [nM]
Venetoclax only 4.61
Venetoclax only 5.27
156 µM JQ1 1.78
313 µM JQ1 0.08
625 µM JQ1 0.003
1.25 µM JQ1 –
2.5 µM JQ1 –
5 µM JQ1 –
JQ1
Viability, %
Venetoclax (log[M])

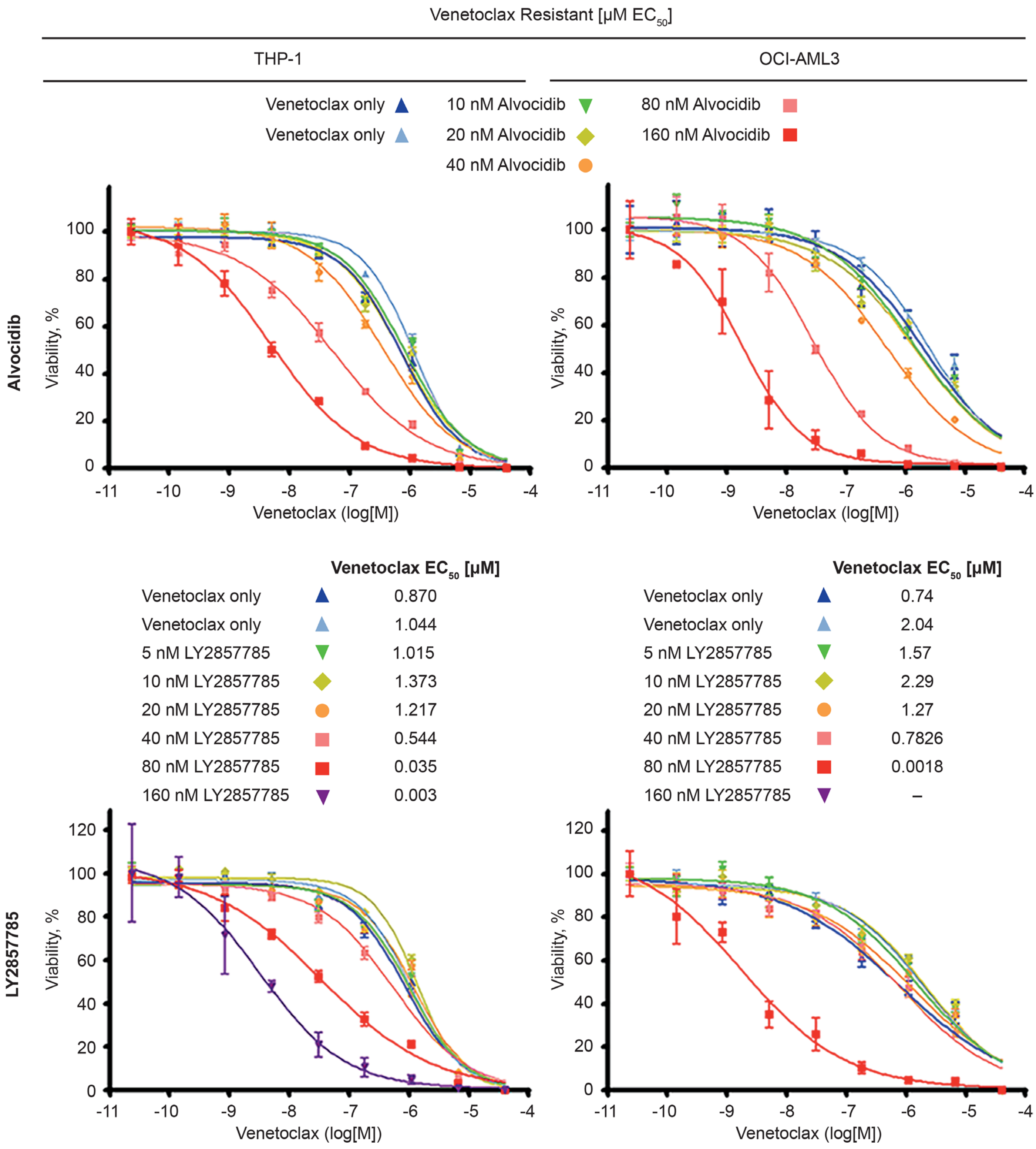
Venetoclax Resistant [μM EC50]
THP-1
OCI-AML3
Venetoclax only
Venetoclax only
10 nM Alvocidib
20 nM Alvocidib
40 nM Alvocidib
80 nM Alvocidib
160 nM Alvocidib
Alvocidib
Viability, %
Venetoclax (log[M])
Venetoclax EC50 [μM]
Venetoclax only 0.870
Venetoclax only 1.044
5 nM LY2857785 1.015
10 nM LY2857785 1.373
20 nM LY2857785 1.217
40 nM LY2857785 0.544
80 nM LY2857785 0.035
160 nM LY2857785 0.003
Venetoclax EC50 [μM]
Venetoclax only 0.74
Venetoclax only 2.04
5 nM LY2857785 1.57
10 nM LY2857785 2.29
20 nM LY2857785 1.27
40 nM LY2857785 0.7826
80 nM LY2857785 0.0018
160 nM LY2857785 –
LY2857785

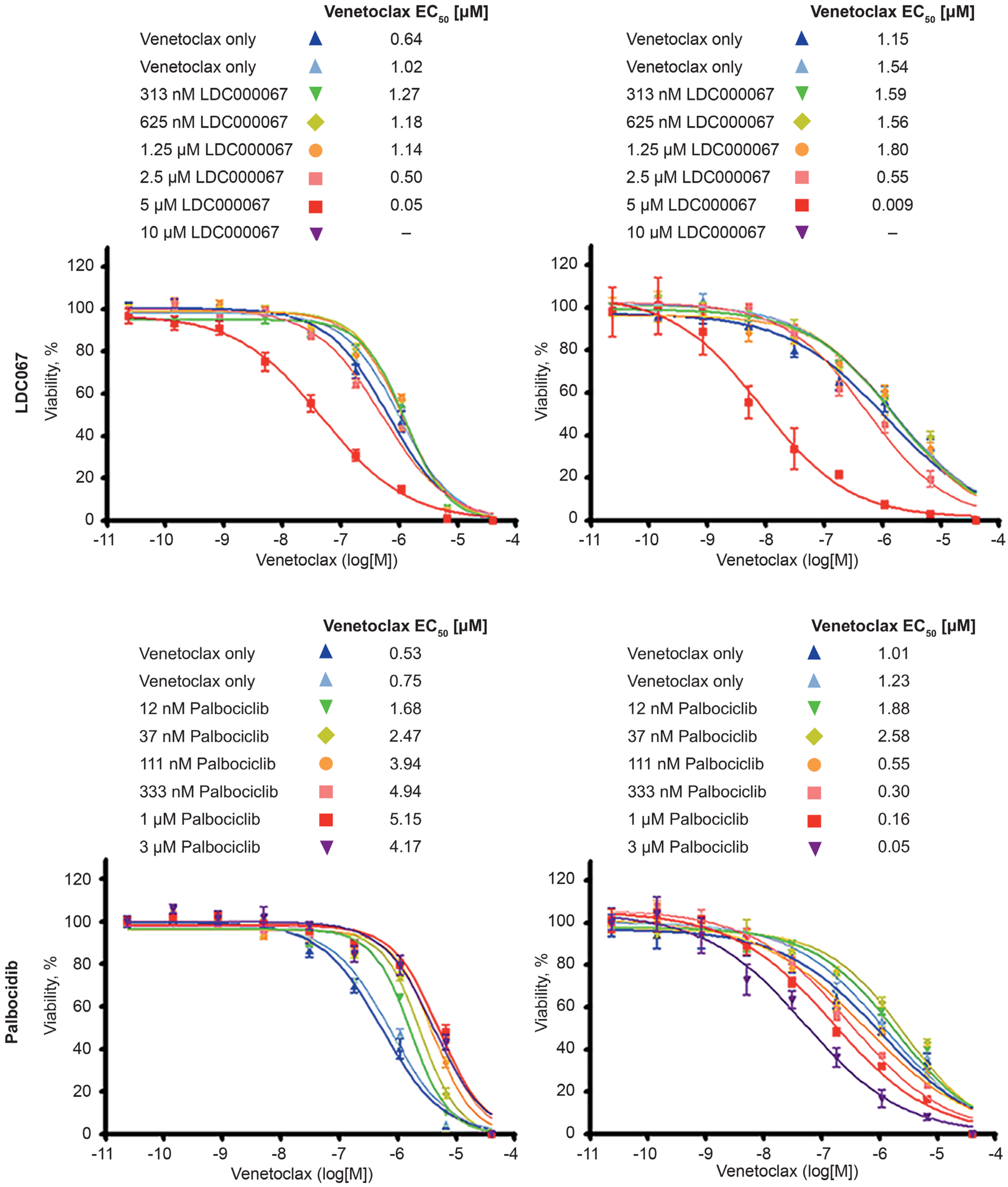

Venetoclax EC50 [µM]
Venetoclax only 0.64
Venetoclax only 1.02
313 nM LDC000067 1.27
625 nM LDC000067 1.18
1.25 µM LDC000067 1.14
2.5 µM LDC000067 0.50
5 µM LDC000067 0.05
10 µM LDC000067 –
LDC067
Viability, %
Venetoclax (log[M])
Venetoclax EC50 [µM]
Venetoclax only 1.15
Venetoclax only 1.54
313 nM LDC000067 1.59
625 nM LDC000067 1.56
1.25 µM LDC000067 1.80
2.5 µM LDC000067 0.55
5 µM LDC000067 0.009
10 µM LDC000067 –
Viability, %
Venetoclax (log[M])
Venetoclax EC50 [µM]
Venetoclax only 0.53
Venetoclax only 0.75
12 nM Palbociclib 1.68
37 nM Palbociclib 2.47
111 nM Palbociclib 3.94
333 nM Palbociclib 4.94
1 µM Palbociclib 5.15
3 µM Palbociclib 4.17
Palbocidib
Viability, %
Venetoclax (log[M])
Venetoclax EC50 [µM]
Venetoclax only 1.01
Venetoclax only 1.23
12 nM Palbociclib 1.88
37 nM Palbociclib 2.58
111 nM Palbociclib 0.55
333 nM Palbociclib 0.30
1 µM Palbociclib 0.16
3 µM Palbociclib 0.05
Viability, %
Venetoclax (log[M])

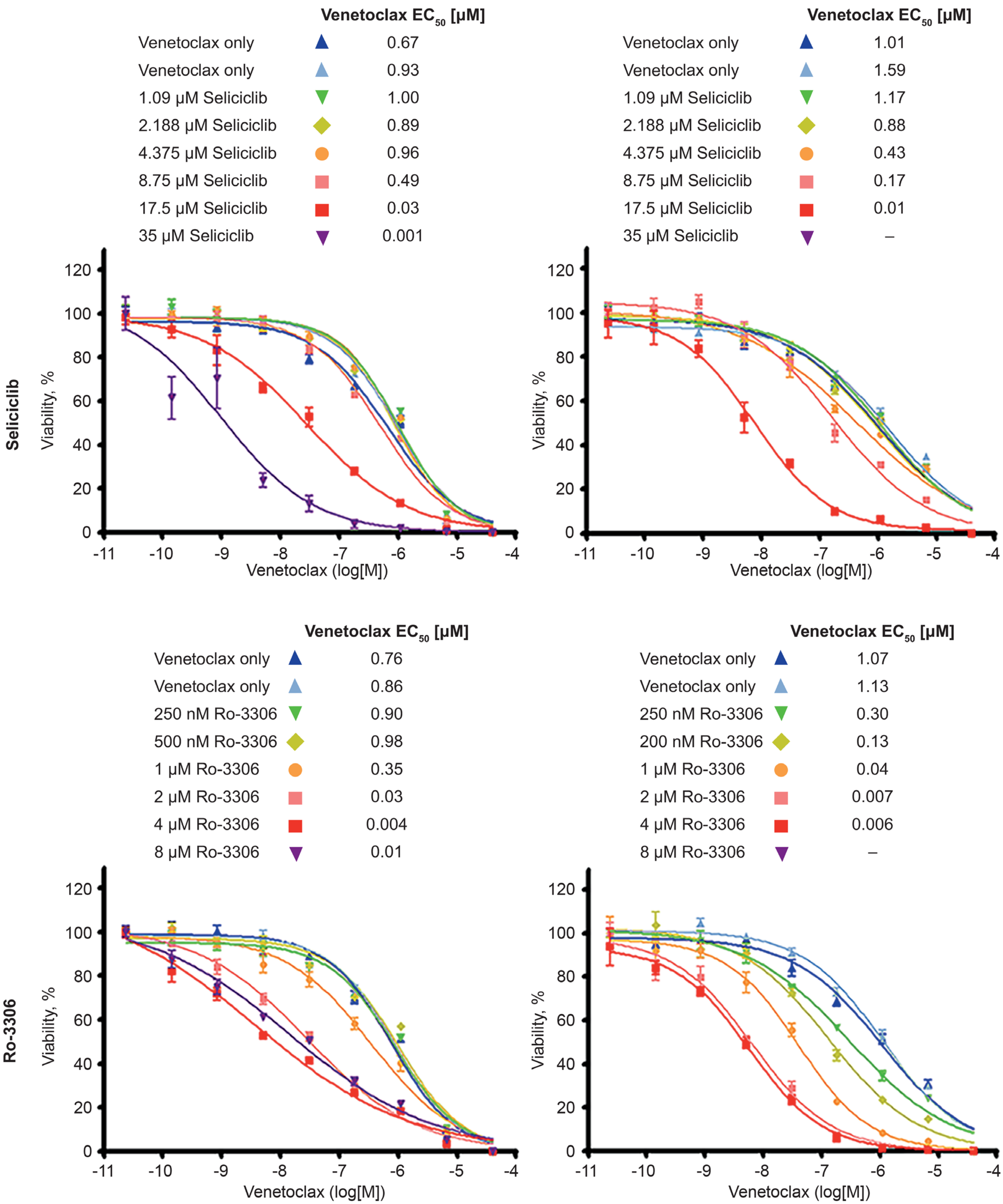
Venetoclax EC50 [μM]
Venetoclax only 0.67
Venetoclax only 0.93
1.09 μM Seliciclib 1.00
2.188 μM Seliciclib 0.89
4.375 μM Seliciclib 0.96
8.75 μM Seliciclib 0.49
17.5 μM Seliciclib 0.03
35 μM Seliciclib 0.001
Venetoclax EC50 [μM]
Venetoclax only 1.01
Venetoclax only 1.59
1.09 μM Seliciclib 1.17
2.188 μM Seliciclib 0.88
4.375 μM Seliciclib 0.43
8.75 μM Seliciclib 0.17
17.5 μM Seliciclib 0.01
35 μM Seliciclib –
Seliciclib
Viability, %
Venetoclax (log[M])
Venetoclax EC50 [μM]
Venetoclax only 0.76
Venetoclax only 0.86
250 nM Ro-3306 0.90
500 nM Ro-3306 0.98
1 μM Ro-3306 0.35
2 μM Ro-3306 0.03
4 μM Ro-3306 0.004
8 μM Ro-3306 0.01
Venetoclax EC50 [μM]
Venetoclax only 1.07
Venetoclax only 1.13
250 nM Ro-3306 0.30
200 nM Ro-3306 0.13
1 μM Ro-3306 0.04
2 μM Ro-3306 0.007
4 μM Ro-3306 0.006
8 μM Ro-3306 –
Ro-3306
Viability, %
Venetoclax (log[M])

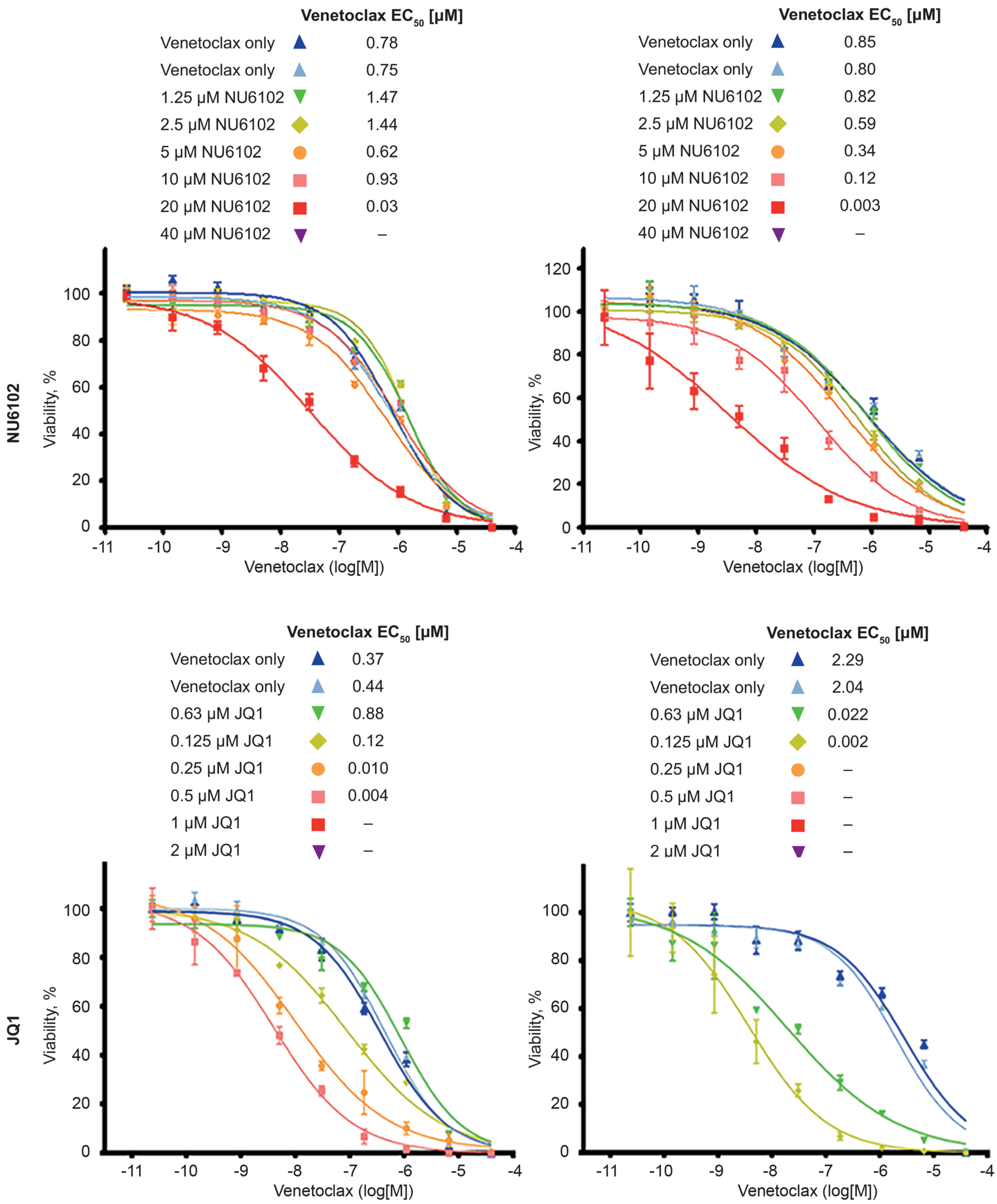

Supplementary Figure 3: Pharmacological dissection of CDK isoforms contributing to Venetoclax sensitization. Combination drug dose response assays were performed with Venetoclax and the inhibitor indicated on the left of each row. Leftward shifts towards lower doses of Venetoclax demonstrate dose-dependent Venetoclax sensitization, while rightward shifts demonstrate Venetoclax antagonism. Venetoclax EC_{50} values from representative experiments are shown as single-agent, and with ascending concentrations of each inhibitor tested, in the key located in the upper right corner of each dose response graph for each cell line (except for alvocidib which is presented in the main text).

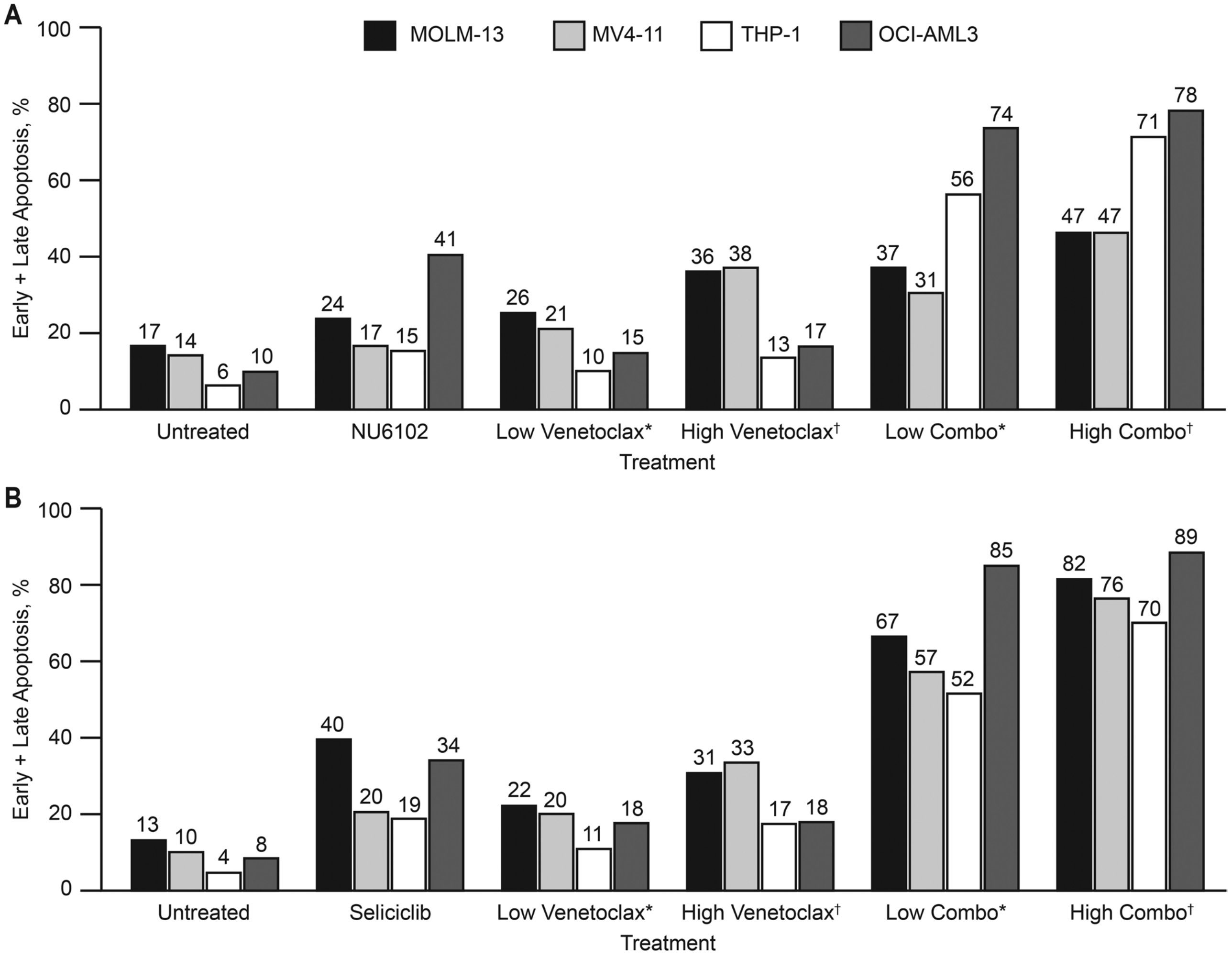

Supplementary Figure 4: Increased apoptosis with combined CDK inhibition and BCL-2 inhibition with Venetoclax. The AML cell lines indicated at the top were treated for 24 hours with 2.5 µM NU6102 for Venetoclax-sensitive cells (MOLM-13 and MV4-11) or 20 µM NU6102 for Venetoclax-resistant cells (OCI-AML3 and THP-1) (**A**), or 17.5 µM seliciclib (**B**), alone and in combination with low dose or high dose of Venetoclax (*2.5 and †10 nM Venetoclax for Venetoclax-sensitive cells, and *0.25 and †1 µMVenetoclax for Venetoclax-resistant cell lines), prior to harvesting for flow cytometry quantification of Annexin V and propidium iodide permeability as a measurement of apoptosis. The percentage of the population in combined early or late apoptosis is plotted on the y-axis.

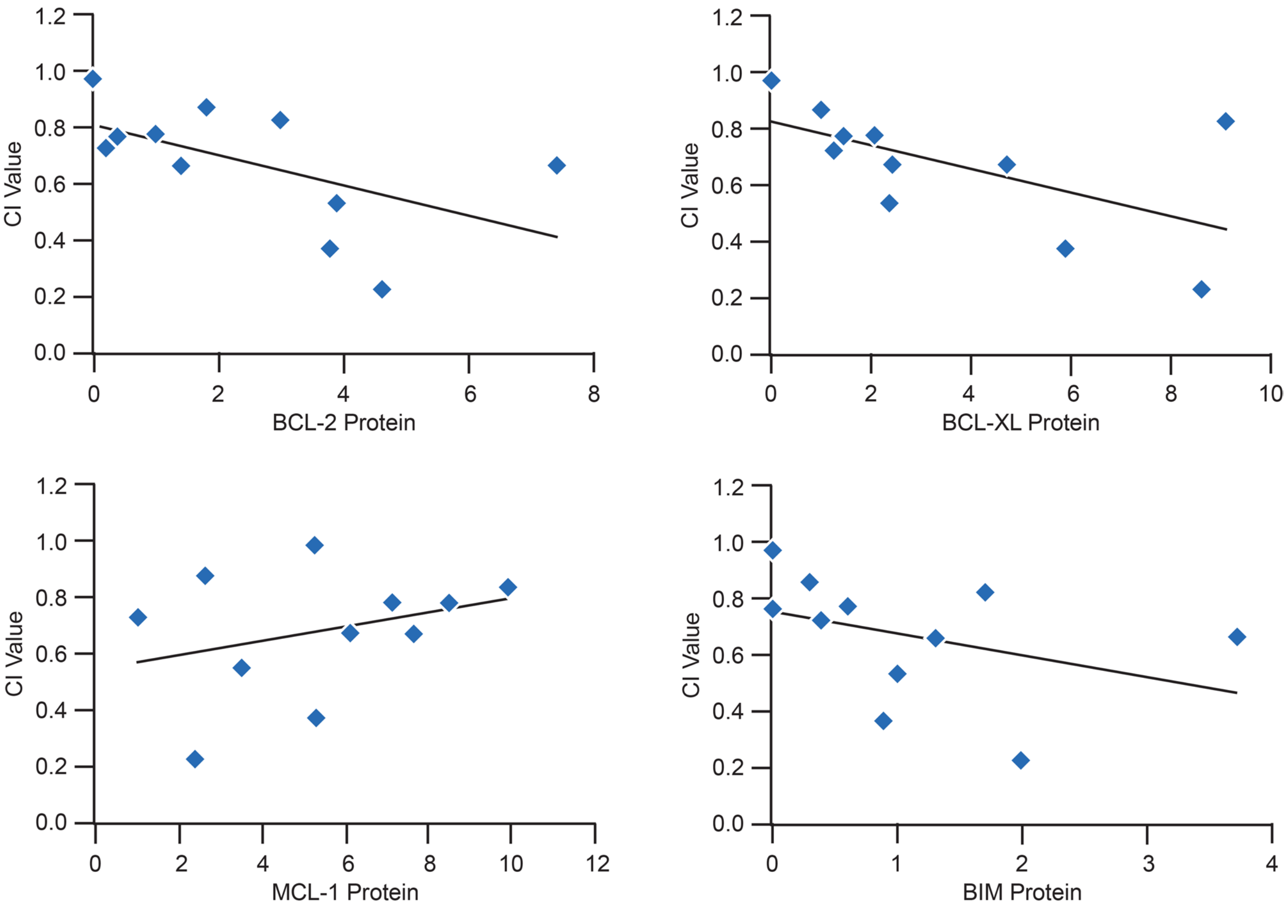

	Combination Index vs. Protein Level	
	R^2	P
BCL-2	0.3409	0.059
BCL-XL	0.3178	0.071
MCL-1	0.0979	0.349
BIM	0.1514	0.264

Supplementary Figure 5: Venetoclax and alvocidib synergy does not significantly correlate with protein levels of individual BCL-2family proteins in primary patient samples. CI values (a metric of Venetoclax/alvocidib synergy) as shown and calculated for primarypatients samples in Figure 5B are plotted against protein levels of the BCL-2 family members (shown on the x-axis) quantifiedin untreated cell lysates from these same AML patient samples, as shown and calculated for Figure 5C. Regression analysis wasused to determine R^2 and *P* values.

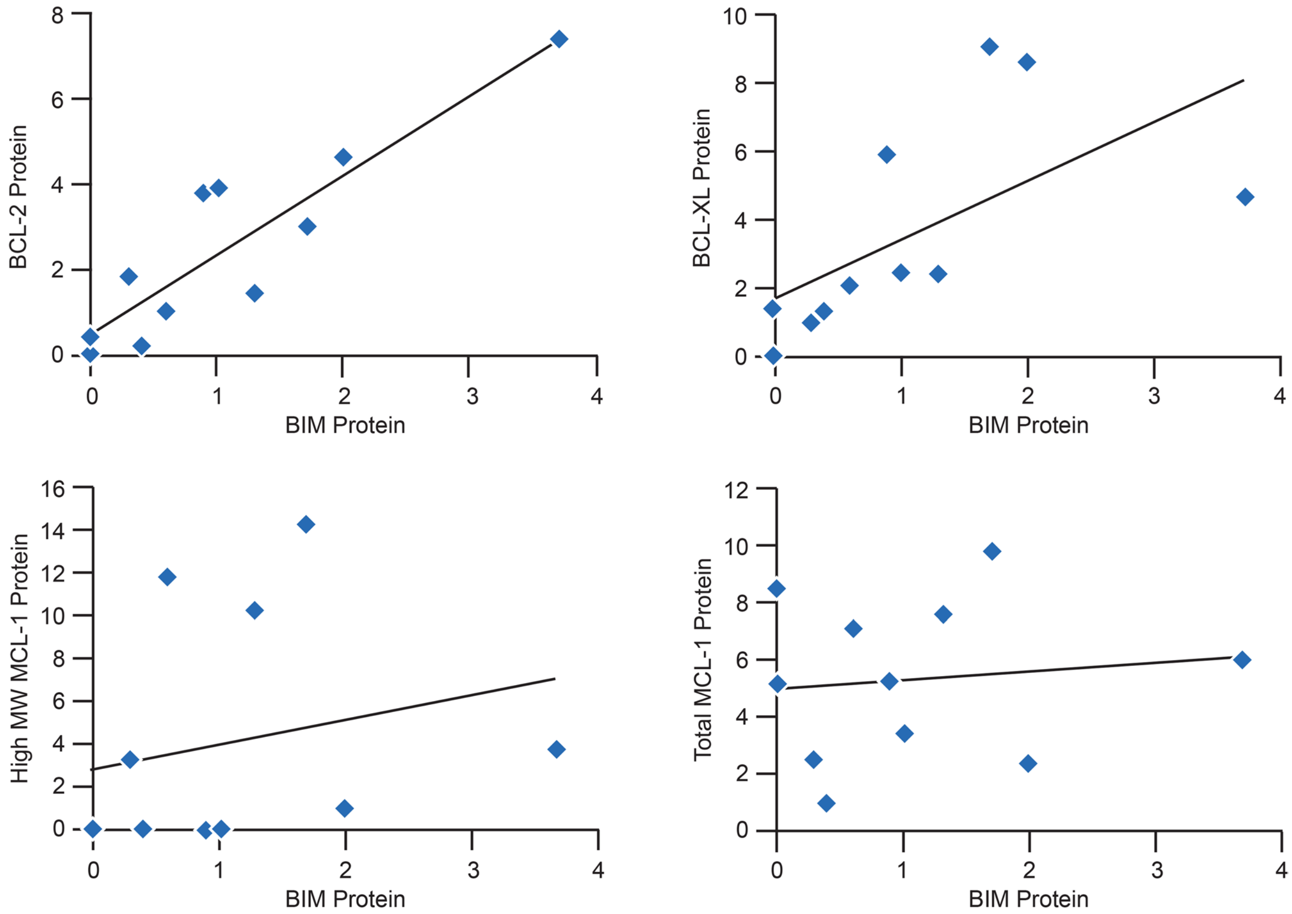

	BIM Protein Level vs. Anti-Apoptotic Protein Level	
	R^2	P
BCL-2	0.8052	1.8E-04
BCL-XL	0.3819	4.3E-02
Total MCL-1	0.0144	7.3E-01
High MW MCL-1	0.0544	4.9E-01

Supplementary Figure 6: Correlation of protein levels of pro-apoptotic BIM versus anti-apoptotic BCL-2, BCL-XL and MCL-1. Protein levels, as shown and calculated for Figure 5C, for the anti-apoptotic BCL-2 family members (indicated on the y-axis) are plotted against BIM protein levels (x-axis). Regression analysis was used to determine R^2 and *P* values.

Supplementary Table 1: Clinical characteristics of primary samples examined ex vivo

Sample #	Diagnosis & treatment history	% Blasts	Mutations and cytogenetics
Sample #1	AML - refractory to 7+3	4	FLT3neg., NPM1-neg., complex karyotype, FISH: MECOM (25%)
Sample #2	AML- de novo	95	FLT3neg., NPM1neg., FISH: +13 (19%), RUNX11 (93%)
Sample #3	AML - refractory to 7+3, on 4th cycle azacitidine	95	FLT3pos., NPM1pos., t(6;17)
Sample #4	AML – antecedent MDS, residual disease after 7+3	5	FLT3pos., t(3;11), FISH: normal
Sample #5	AML- de novo	23	FLT3neg., NPM1neg., FISH: normal
Sample #6	AML – history of lupus/TNF-α	63	FLT3-ITDpos., NPM1pos., BCR-ABLneg., FISH: normal
Sample #7	AML- refractory to azacitidine	96	FLT3-ITDpos., MLL-PTDpos., TET2, DNMT3a
Sample #8	AML- de novo	43	FLT3pos., NPM1neg., inv16
Sample #9	AML – antecedent MDS, 4x prior therapies: quizartinib, pinometostat, decitabine, sorafenib	N/A	FLT3-ITDpos., MLL dup., FISH: +8(4%)
Sample #10	AML- de novo, relapse after stem cell transplant	96	FLT3-ITDpos., NPM1pos., t(8;15), FISH: MECOM (30%)
Sample #11	AML in remission post-ACM regimen	1	BM collected due to cytopenias
Sample #12	AML - de novo, early relapse, high-dose cytarabine, 2 cycles azacitidine	87	FLT3-ITDpos., FISH: normal
Sample #13	AML- refractory to azacitidine	70	FISH: +8(40%), MECOM (4%)
Sample #14	AML with myelodysplastic changes	42	Complex karyotype, FLT3neg.
